# Long-term exposure to ambient air pollutants and female infertility risk: a population-based cohort study in Taiwan

**DOI:** 10.1186/s12889-025-24213-x

**Published:** 2025-09-30

**Authors:** Yu-Chieh Lo, Yeu-Chai Jang, Shu-Han Chuang, Shun-Jen Cheng, Yi-Jie Kuo, Cheng-Hsien Chang, Yu-Pin Chen

**Affiliations:** 1 Department of Orthopedics, Taichung Veterans General University, Taichung, Taiwan; 2 Department of Obstetrics and Gynecology, Wan Fang Hospital, Taipei Medical University, Taipei, Taiwan; 3 Division of General Practice, Department of Medical Education, Changhua Christian Hospital, Changhua, Taiwan; 4 Department of Orthopedics, Wan Fang Hospital, Taipei Medical University, Taipei, Taiwan; 5 Department of Orthopedics, School of Medicine, College of Medicine, Taipei Medical University, Taipei, Taiwan; 6 Department of Ophthalmology, Changhua Christian Hospital, Changhua, Taiwan; 7 Department of Post-Baccalaureate Medicine, College of Medicine, National Chung Hsing University, Taichung, Taiwan

**Keywords:** Female infertility, Air pollution, Reproductive health

## Abstract

**Introduction:**

Infertility affects over 186 million people globally, with about 1 in 7 couples in developed nations experiencing it. Causes include age-related fertility decline and environmental factors. Air pollution is a potential factor, but large-scale evidence is still lacking. This study examines the impact of several air pollutants on infertility in females aged 15 to 60, hypothesizing that air pollution increases infertility risks.

**Method:**

We constructed a cohort in Taiwan between July 1, 2003, and December 31, 2013, using the National Health Insurance Research Database (NHIRD) of females aged 15 to 60. Concentrations of SO2, CO, O3, PM10, PM2.5, NOX, NO, NO2, THC, NMHC, and CH4 were estimated based on insurance registration. We calculated the HRs of exposure at a standard deviation increment for 10 years to determine the dose–response effect between air pollutants and infertility.

**Result:**

Long-term exposure to SO2, CO, PM10, PM2.5, NOX, NO, NO2, THC, NMHC, and CH4 was associated with increased infertility in women of reproductive age. Each standard deviation increase in exposure to these pollutants was associated with a higher incidence of infertility by 13%, 116%, 35%, 77%, 80%, 66%, 76%, 116%, 52%, and 181%, respectively. Conversely, ozone exposure was associated with a 52% reduction in infertility risk.

**Conclusion:**

This study demonstrates the significant impact of air pollution on female infertility, showing a clear dose–response relationship between exposure to various pollutants and infertility rates. These findings highlight the need for efforts to reduce air pollution and its effects on reproductive health. Further research is needed to understand the mechanisms and inform public health policies.

**Supplementary Information:**

The online version contains supplementary material available at 10.1186/s12889-025-24213-x.

## Introduction

Female infertility refers to the incapacity to achieve a clinical pregnancy, impacting more than 186 million individuals globally [1]. It is estimated to affect approximately 1 in 7 couples in developed nations and about 8–12% of reproductive-aged couples worldwide [1]. The World Health Organization defines infertility as the inability to achieve a clinical pregnancy after 12 months or more of regular unprotected sexual intercourse [2]. Numerous factors that could affect the inherent fertility of couples have been acknowledged. These encompass the duration of undesired non-conception, the decline in female fertility associated with age, infertility attributed to medical conditions, sperm quality, the influence of endocrine-disrupting chemicals, and environmental origins [3].

Air pollution emerges as a significant health concern, correlating with heightened rates of both mortality and morbidity [4]. For example, it has been linked to elevated cancer risk, as well as cardiovascular and respiratory disorders in both adults and children [5]. Furthermore, it's associated with negative perinatal outcomes and associated with increased mortality following hip fracture in older adults [6]. Several lines of evidence suggest that prenatal exposure to environmental pollutants may influence reproductive and endocrine health, with emerging research linking maternal exposure from passive smoking and traffic-related air pollution to alterations in neonatal hormonal profiles [7]. Two systematic reviews indicate a strong link between female infertility and air pollution [8]. Conforti et al. found that in the IVF population, nitrogen dioxide and ozone were associated with reduced live birth rates, while in the general population, particulate matter (PM2.5 and PM10), sulfur dioxide, carbon monoxide, and nitrogen dioxide were linked to reduced fecundability, miscarriage, and stillbirths due to air pollution exposure [8]. Another systematic review analyzed the impact of air pollution on fertility rates, observing a statistically significant reduction in fertility rates associated with higher traffic-related air pollution, particularly in relation to the coarse fraction of particulate matter (PM) [9]. Apart from the aforementioned study, animal research has explored the impact of particulate urban air pollution (PM 2.5) on reproductive health. These studies found that exposure to PM 2.5 is associated with a reduced number of viable fetuses, higher incidence of implantation failures, low birth weight, low placenta weight, changes in estrous cyclicity, decreased ovarian follicles count, and decreased fertility indices [10].

Given the paucity of clinical evidence regarding the impact of air pollutants on female infertility, we embarked on this retrospective study to investigate the potential influence of decade-long exposure to various air pollutants on the likelihood of infertility among Taiwanese women aged 15 to 60. Our hypothesis postulated that extended exposure to air pollution might affect the incidence of female infertility. Specifically, we examined the effects of 11 air pollution compounds: sulfur dioxide (SO2), carbon monoxide (CO), ozone (O3), particulate matter with a diameter of less than 10 μm (PM10), particulate matter with a diameter of less than 2.5 μm (PM2.5), nitrogen oxides (NOX), nitrogen monoxide (NO), nitrogen dioxide (NO2), total hydrocarbons (THC), nonmethane hydrocarbons (NMHC), and methane (CH4). Through the utilization of a population-based approach and comprehensive air pollutant exposure data, this study endeavors to bridge these significant knowledge gaps, offering insights into the intricate relationship between air pollution and the risk of female infertility.

## Materials and methods

### Data source

In this study, we retrieved data from the National Health Insurance Research Database (NHIRD) of Taiwan, which was launched by the Taiwan government in 1995 and collected comprehensive health care for 98.29% of the population [11]. The database provided nationwide medical information including gender, date of birth, employment, inpatient and outpatient diagnoses, medical procedures, drug usage, treatment duration, and medical costs of approximately 23 million insured individuals in Taiwan [12]. We identified baseline information by using the Longitudinal Health Insurance Database 2000, a subset of the NHIRD containing 1 million randomly enrolled patients. The Research Ethics Committee approved this study protocol (IRB: 240310). This study was conducted in accordance with the principles of the Declaration of Helsinki and relevant regulations. Due to the anonymity of patient data in NHIRD, patient-informed consent was not required for this study.

### Study population

We only included patients with complete available data, while those with missing, inconsistent, or unknown records of baseline information such as gender and birth year were therefore precluded. The enrollment population was defined as those who were female gender between July 1, 2003, and December 31, 2013. The further criteria for exclusion were detailed as follows, 1) subjects aged under 15 or over 60 at the beginning of the study, 2) those diagnosed with infertility, history of prior surgery of the genital organs, or history of radiotherapy before the beginning of the study, 3) subjects whose survival data were before the beginning of the study, and 4) subjects’ follow-up were under 5 years. To ensure adequate accumulation of long-term exposure and reliable outcome ascertainment, only participants with at least 5 years of follow-up were included. This threshold improves data stability and reduces the likelihood of misclassification.

### Exposure data collection

We obtained the cumulative daily average concentration of eleven air pollutants, including sulfur dioxide (SO_2_), carbon monoxide (CO), ozone (O_3_), particulate matter < 10 μm in size (PM_10_), particulate matter < 2.5 μm in size (PM_2.5_), nitrogen oxides (NO_X_), nitrogen monoxide (NO), nitrogen dioxide (NO_2_), total hydrocarbons (THC), nonmethane hydrocarbons (NMHC), and methane (CH4), from the measurements recorded by the 76 monitoring stations maintained by the Taiwan Environmental Protection Administration (EPA), Executive Yuan. Data were collected for the period from July 1, 1993, to December 31, 2013. The cumulative daily average concentration of each pollutant was calculated from 10 years prior to the end of follow-up. Daily concentrations were extracted from the nearest monitoring station linked to each residence, and a time-weighted average was calculated across all registered addresses during this period. To account for residential mobility during the study period, we linked the air pollutant concentrations to each individual’s residential postal codes based on their insurance registration records. For participants who changed residence, exposure levels were updated accordingly, and the cumulative exposure was calculated as a time-weighted average based on the duration spent at each registered location.

### Outcomes and confounders

The development of infertility was the major outcome of interest. Infertility diagnosis was based on ICD-9-CM codes 628.x, confirmed by ≥ 2 outpatient visits or ≥ 1 inpatient claim. We followed every patient until the primary end-point defined by either the development of infertility (withdrawal from the NHI program) or end of the study period (December 31, 2013), with survival time measured in months. Several confounders were introduced and adjusted in this study, including age, urbanization level, insurance amount, CCI score, ambient temperature, season, and lag0-2. Above them, patient data such as age, insurance amount, and CCI score were obtained from the NHIRD database. Specifically, the insurance amount was measured as the average value during the period of air pollutant exposure assessment, while the comorbidities were defined before the survival date. The meteorological factor of ambient temperature was collected from the EPA. The urbanization level was recorded according to the patient’s residence at the beginning of the follow-up period, while the season was defined by date. We also adopted the 3-day moving average of current day and two preceding days concentrations of air pollutants before the primary end-point (lag0-2) for adjusted confounders, based on the largest effect estimate found in previous literature [13]. Confounders were selected based on literature linking them to infertility and availability in the NHIRD. Lifestyle-related variables not directly available (e.g., smoking, BMI) were partially captured through insurance income, urbanization, and comorbidity proxies.

### Statistical analysis

To demonstrate patient characteristics associated with air pollution, patients were divided by the exposure concentration of each pollutant into 3 tertiles and were compared using the chi-square chi-squared test or one-way analysis of variance among tertiles, post hoc tests were performed to explore differences when significance was indicated in the one-way analysis of variance. We plotted crude cumulative incidence curves of infertility for participants in three tertiles, with the difference between tertiles assessed by log-rank tests. We conducted Cox regression models to explore the dose–response effect between air pollutants and infertility risk by calculating the HRs of exposure at a standard deviation increment for 10 years. The regression models were adjusted for the abovementioned confounding factors. To address potential confounding from multiple coexisting pollutants, we included additional pollutants with low correlation (*r* < 0.3) to the primary exposure in the adjusted models. This reduces confounding without introducing multicollinearity. We also constructed two-pollutant models to test the robustness of the observed associations and assess the independent effect of each pollutant. All tests were 2-sided, and statistically, significance was considered when *p* < 0.05. All analyses were performed using the MetaTrial Research Platform. The software was developed by Biomedica Corporation, Taiwan.

## Results

### Study population

**Fig. 1:**
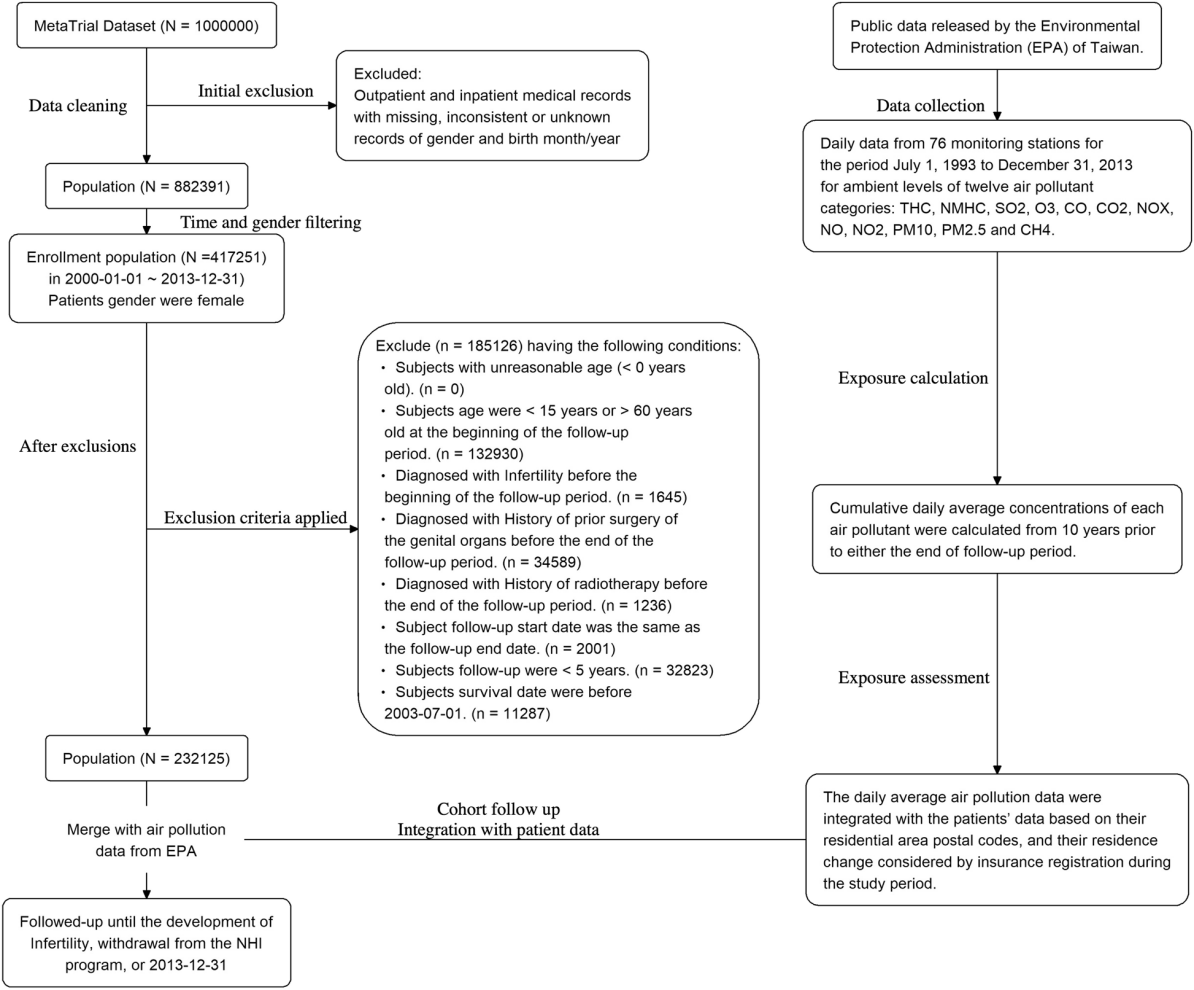
The flow diagram of the selection process

After the inclusion of patients with complete available data (*n* = 882391), those who were female gender were identified (*n* = 417251). The following exclusion criteria were then applied: (1) age under 15 or over 60 years at study initiation (*n* = 132,930); (2) diagnosis of infertility, history of prior genital organ surgery, or history of radiotherapy before study initiation (*n* = 1,645); (3) participants who died before the study start date (*n* = 35,825); and (4) follow-up duration under 5 years (*n* = 32,823). Consequently, 232,125 participants were included in the final analysis, with the selection process illustrated in Fig. 1.

### Characteristics and descriptive results

Table 1 presents the characteristics of the included cohort (*n* = 232,125), among whom 929 individuals (12.51%) were diagnosed with infertility during the follow-up period. The distribution of comorbidities is also shown. Characteristics stratified by tertiles of pollutant exposure are detailed in Supplementary Table 1, while mean concentrations and distributions of 10-year pollutant exposures are presented in Table 2.

**Table 1:** Baseline characteristics

Characteristic	N (%)
Infertility	4981 (2.15)
Age, years	
15–35	121732 (52.44)
36–60	110393 (47.56)
Mean ± SD	35.08 ± 12.45
Urbanization level	
1 (highest)	138881 (59.83)
2	71156 (30.65)
3	14280 (6.15)
4 (lowest)	1826 (0.79)
unknown	5982 (2.58)
Insurance amount, NT$	
financially dependent	1343 (0.58)
1–19999	79868 (34.41)
20000–39999	102875 (44.32)
> = 40000	41027 (17.67)
unknown	7012 (3.02)
CCI score	
Mean ± SD	1.10 ± 1.68
Comorbidity	
Inflammatory disease of the ovary, fallopian tube, pelvic cellular tissue, and peritoneum	35926 (15.48)
Inflammatory disease of the uterus	12241 (5.27)
Inflammatory disease of the cervix, vagina, and vulva	107803 (46.44)
Endometriosis	8828 (3.80)
Hypertension	38765 (16.70)
Diabetes mellitus	27325 (11.77)
Hypertriglyceridemia	286 (0.12)
Hypercholesterolemia	29970 (12.91)
Coronary artery disease	21789 (9.39)
Disorders of eating	302 (0.13)

*SD* standard deviation, *CCI* score, Charlson Comorbidity Index score

**Table 2:** Mean and distribution of air pollutants over the exposure period

	SO_2_ (ppb)	CO (ppm)	O_3_ (ppb)	PM_10_ (μg/m^3^)	PM_2.5_ (μg/m^3^)	NO_X_ (ppb)	NO (ppb)	NO_2_ (ppb)	THC (ppm)	NMHC (ppm)	CH_4_ (ppm)
Mean	4.14	0.53	28.67	54.19	32.43	25.72	7.54	18.18	2.24	0.29	1.95
SD	1.35	0.11	1.73	8.93	6.05	6.8	3.68	3.48	0.12	0.08	0.07
T_1_	3.59	0.48	27.77	47.66	27.67	21.44	4.93	16.55	2.17	0.25	1.93
T_2_	4.04	0.59	29.11	56.18	34.95	29.6	8.51	20.49	2.31	0.33	1.97

*SD* standard deviation, *T*_*1*_ 33.33 percentile, *T*_*2*_ 66.66 percentile, *ppb* parts per billion; ppm, parts per million, *μg/m*^*3*^ microgram/cubic meter, *SO*_*2*_ sulfur dioxide, *CO* carbon monoxide, *O*_*3*_ ozone, *PM*_*10*_ particulate matter < 10 μm in size, *PM*_*2.5*_ particulate matter < 2.5 μm in size, *NO*_*X*_ nitrogen oxides, *NO* nitrogen monoxide, *NO*_*2*_ nitrogen dioxide, THC total hydrocarbons, *NMHC* nonmethane hydrocarbons, *CH*_*4*_ methane

### Cumulative incidence of infertility

**Fig. 2:**
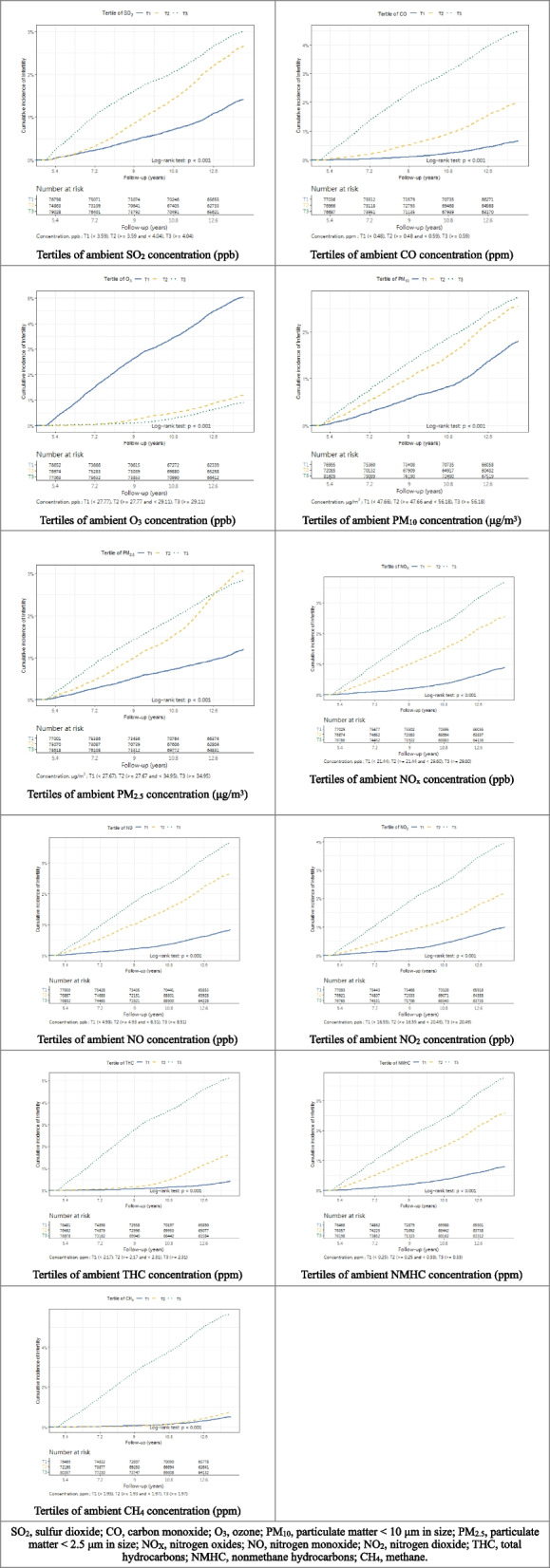
Cumulative incidence curves of infertility stratified by tertiles of air pollutant exposure concentrations

For the outcome of interest, the incidence of infertility, we exhibited the results among tertiles in Table 3, with the *p*-value of one-way analysis of variance and post-hoc analysis. The cumulative incidence curves (Fig. 2) illustrate variation in infertility occurrence across different exposure levels, suggesting pollutant-specific patterns.

**Table 3:** Incidence of infertility among tertiles

Pollutants	Tertiles of average daily exposure, n (%)			*P* value	Total, n (%)
	T1 (lowest)	T2	T3 (highest)		
SO_2_	974/77141 (1.26)	1799/75237 (2.39)	2208/79747 (2.77)	< 0.001	4981/232125 (2.15)
CO	443/77375 (0.57)	1351/77375 (1.75)	3187/77375 (4.12)	< 0.001	4981/232125 (2.15)
O_3_	3579/77372 (4.63)	796/77365 (1.03)	606/77388 (0.78)	< 0.001	4981/232125 (2.15)
PM_10_	1243/77360 (1.61)	1681/72522 (2.32)	2057/82243 (2.50)	< 0.001	4981/232125 (2.15)
PM_2.5_	832/77369 (1.08)	2077/75510 (2.75)	2064/79229 (2.61)	< 0.001	4973/232108 (2.14)
NO_X_	603/77370 (0.78)	1778/77380 (2.30)	2600/77375 (3.36)	< 0.001	4981/232125 (2.15)
NO	561/77375 (0.73)	1839/77374 (2.38)	2581/77376 (3.34)	< 0.001	4981/232125 (2.15)
NO_2_	675/77348 (0.87)	1506/77402 (1.95)	2800/77375 (3.62)	< 0.001	4981/232125 (2.15)
THC	268/76816 (0.35)	1084/76816 (1.41)	3608/76817 (4.70)	< 0.001	4960/230449 (2.15)
NMHC	541/76813 (0.70)	1786/76819 (2.32)	2633/76817 (3.43)	< 0.001	4960/230449 (2.15)
CH_4_	335/76816 (0.44)	451/72513 (0.62)	4174/81120 (5.15)	< 0.001	4960/230449 (2.15)

*SO*_*2*_ sulfur dioxide, *CO* carbon monoxide, *O*_*3*_ ozone, *PM*_*10*_ particulate matter < 10 μm in size, *PM*_*2.5*_ particulate matter < 2.5 μm in size, *NO*_*X*_ nitrogen oxides, *NO* nitrogen monoxide, *NO*_*2*_ nitrogen dioxide, *THC* total hydrocarbons, *NMHC* nonmethane hydrocarbons, *CH*_*4*_ methane

### The dose–response effect between air pollutants and the incidence of infertility

To explore the dose–response effect between the concentrations of air pollutants and the incidence of infertility, we performed Cox regression models and calculated the HRs of exposure at a standard deviation increment for 10 years. The adjusted hazard ratios (HRs) are listed in Table 4. Each SD increase in the average exposure concentration of SO₂, CO, PM₁₀, PM₂.₅, NOX, NO, NO₂, THC, NMHC, and CH₄ was associated with a significantly elevated hazard of infertility by 13%, 116%, 35%, 77%, 80%, 66%, 76%, 116%, 52%, and 181%, respectively. In contrast, O₃ exposure was negatively associated with infertility, with a 52% lower hazard (HR: 0.48), indicating a statistically significant protective trend. To further illustrate the dose–response relationship, we categorized the exposure concentrations into tertiles and conducted Cox regression analyses using the lowest tertile (T1) as the reference. The adjusted hazard ratios for the second (T2) and third (T3) tertiles are presented in Table 5. A consistent trend of increasing hazard of infertility was observed across tertiles for most pollutants, particularly SO₂, CO, PM₁₀, PM₂.₅, NOX, NO, NO₂, THC, NMHC, and CH₄. Conversely, higher O₃ exposure tertiles were associated with a significantly reduced hazard, reinforcing the negative relationship seen in the standard deviation-based model.

**Table 4:** Hazard ratios for incidence of infertility of long-term exposure at an SD increment

Pollutant	Adjusted HR (95% CI)	*P* value	SD
SO_2_	1.13 (1.09,1.18)	< 0.001	1.13 ppb
CO	2.16 (2.09,2.22)	< 0.001	0.11 ppm
O_3_	0.48 (0.47,0.49)	< 0.001	1.73 ppb
PM_10_	1.35 (1.28,1.43)	< 0.001	8.93 μg/m^3^
PM_2.5_	1.77 (1.67,1.88)	< 0.001	6.05 μg/m^3^
NO_X_	1.80 (1.73,1.86)	< 0.001	6.80 ppb
NO	1.66 (1.61,1.72)	< 0.001	3.68 ppb
NO_2_	1.76 (1.69,1.83)	< 0.001	3.48 ppb
THC	2.16 (2.09,2.22)	< 0.001	0.12 ppm
NMHC	1.52 (1.47,1.56)	< 0.001	0.08 ppm
CH_4_	2.81 (2.72,2.90)	< 0.001	0.07 ppm

Cox regression models were adjusted for age, urbanization level, insurance amount, CCI score, Inflammatory disease of the ovary, fallopian tube, pelvic cellular tissue, and peritoneum, Inflammatory disease of the uterus, Inflammatory disease of the cervix, vagina, and vulva, Endometriosis, Hypertension, Diabetes mellitus, Hypertriglyceridemia, Hypercholesterolemia, Coronary artery disease, ambient temperature, lag0-2, season, and controlled pollutants

*HR* hazard ratio, *CI* confidence interval, *SD* standard deviation, *SO*_*2*_ sulfur dioxide, *CO* carbon monoxide, *O*_*3*_ ozone, *PM*_*10*_ particulate matter < 10 μm in size, *PM*_*2.5*_ particulate matter < 2.5 μm in size, *NO*_*X*_ nitrogen oxides, *NO* nitrogen monoxide, *NO*_*2*_ nitrogen dioxide, *THC* total hydrocarbons, *NMHC* nonmethane hydrocarbons, *CH*_*4*_ methane

**Table 5:** The dose–response association between air pollutants and infertility risk

Pollutant category	Tertiles of average daily pollutant	Population	Infertility	PY	Incidence Rate	Adjusted HR (95% CI)	*P* value
SO2	T1 (lowest)	Total (*N* = 232125)	974	1017086	9.58	1.00 (reference)	-
	T2		1799	985662	18.25	1.22 (1.13,1.32)	< 0.001
	T3 (highest)		2208	1036732	21.30	1.71 (1.58,1.86)	< 0.001
CO	T1 (lowest)	Total (*N* = 232125)	443	1022715	4.33	1.00 (reference)	-
	T2		1351	1014329	13.32	1.67 (1.50,1.87)	< 0.001
	T3 (highest)		3187	1002435	31.79	3.47 (3.11,3.88)	< 0.001
O3	T1 (lowest)	Total (*N* = 232125)	3579	998055	35.86	1.00 (reference)	-
	T2		796	1017205	7.83	0.30 (0.28,0.33)	< 0.001
	T3 (highest)		606	1024219	5.92	0.24 (0.21,0.26)	< 0.001
PM10	T1 (lowest)	Total (*N* = 232125)	1243	1020533	12.18	1.00 (reference)	-
	T2		1681	948868	17.72	1.36 (1.26,1.47)	< 0.001
	T3 (highest)		2057	1070078	19.22	2.01 (1.82,2.22)	< 0.001
PM2.5	T1 (lowest)	Total (*N* = 232108)	832	1021415	8.15	1.00 (reference)	-
	T2		2077	988029	21.02	1.23 (1.13,1.33)	< 0.001
	T3 (highest)		2064	1029946	20.04	2.25 (2.02,2.51)	< 0.001
NOX	T1 (lowest)	Total (*N* = 232125)	603	1021760	5.90	1.00 (reference)	-
	T2		1778	1009107	17.62	1.69 (1.53,1.86)	< 0.001
	T3 (highest)		2600	1008611	25.78	2.34 (2.11,2.60)	< 0.001
NO	T1 (lowest)	Total (*N* = 232125)	561	1021043	5.49	1.00 (reference)	-
	T2		1839	1009660	18.21	1.72 (1.56,1.90)	< 0.001
	T3 (highest)		2581	1008777	25.59	2.57 (2.30,2.86)	< 0.001
NO2	T1 (lowest)	Total (*N* = 232125)	675	1021060	6.61	1.00 (reference)	-
	T2		1506	1011807	14.88	1.44 (1.31,1.59)	< 0.001
	T3 (highest)		2800	1006612	27.82	2.13 (1.93,2.34)	< 0.001
THC	T1 (lowest)	Total (*N* = 230449)	268	1015554	2.64	1.00 (reference)	-
	T2		1084	1012641	10.70	1.12 (0.97,1.29)	0.132
	T3 (highest)		3608	989019	36.48	3.53 (3.07,4.06)	< 0.001
NMHC	T1 (lowest)	Total (*N* = 230449)	541	1013917	5.34	1.00 (reference)	-
	T2		1786	1003607	17.80	1.30 (1.16,1.45)	< 0.001
	T3 (highest)		2633	999690	26.34	1.85 (1.65,2.08)	< 0.001
CH4	T1 (lowest)	Total (*N* = 230449)	335	1014880	3.30	1.00 (reference)	-
	T2		451	960311	4.70	1.03 (0.89,1.20)	0.670
	T3 (highest)		4174	1042023	40.06	2.99 (2.65,3.38)	< 0.001

*PY* person years, *HR* hazard ratio, *CI* confidence interval, *SO2* sulfur dioxide, *CO* carbon monoxide, *O3* ozone, *PM10* particulate matter < 10 μm in aerodynamic diameter, *PM2.5* particulate matter < 2.5 μm in aerodynamic diameter, *NOX* nitrogen oxides, *NO* nitric oxide, *NO2* nitrogen dioxide, *THC* total hydrocarbons, *NMHC* non-methane hydrocarbon, *CH4* methane

The tertile values, in ppb (SO2, O3, NOX, NO, NO2); ppm (CO, THC, NMHC, CH4); and μg/m3 (PM10, PM2.5) were as follows:

SO2 (T1: < 3.59, T2: ≥ 3.59 and < 4.04, T3: ≥ 4.04); CO (T1: < 0.48, T2: ≥ 0.48 and < 0.59, T3: ≥ 0.59); O3 (T1: < 27.77, T2: ≥ 27.77 and < 29.11, T3: ≥ 29.11); PM10 (T1: < 47.66, T2: ≥ 47.66 and < 56.18, T3: ≥ 56.18); PM2.5 (T1: < 27.67, T2: ≥ 27.67 and < 34.95, T3: ≥ 34.95); NOX (T1: < 21.44, T2: ≥ 21.44 and < 29.60, T3: ≥ 29.60); NO (T1: < 4.93, T2: ≥ 4.93 and < 8.51, T3: ≥ 8.51); NO2 (T1: < 16.55, T2: ≥ 16.55 and < 20.49, T3: ≥ 20.49); THC (T1: < 2.17, T2: ≥ 2.17 and < 2.31, T3: ≥ 2.31); NMHC (T1: < 0.25, T2: ≥ 0.25 and < 0.33, T3: ≥ 0.33); CH4 (T1: < 1.93, T2: ≥ 1.93 and < 1.97, T3: ≥ 1.97)

per 10,000 person-years

To assess age-related confounding, a sensitivity analysis restricted to women aged 15–45 years was performed. Results remained consistent with the primary analysis. For example, PM₂.₅ exposure remained significantly associated with increased infertility hazard (HR: 1.74, 95% CI: 1.61–1.88, *p* < 0.001). These results are presented in Supplementary Tables S4 and S5.

## Discussion

### Principal results

This study found that long-term exposure to SO_2_, CO, PM_10_, PM_2.5_, NO_X_, NO, NO_2_, THC, NMHC, and CH_4_ was associated with the incidence of infertility in age-reproductive women. In females, each increase of a standard deviation in average exposure concentration of SO_2_, CO, PM_10_, PM_2.5_, NO_X_, NO, NO_2_, THC, NMHC, and CH_4_, indicated a higher incidence of infertility of 13%, 116%, 35%, 77%, 80%, 66%, 76%, 116%, 52%, and 181%, respectively. On the contrary, we found a significant reduction of 52% in the hazard ratio with ozone. Given that fertility typically declines after age 40, the inclusion of women aged 46–60 in our main analysis may raise concerns about age-related bias. To address this, we performed a sensitivity analysis focusing on women aged 15–45 years, consistent with the WHO-defined reproductive age range. The results were highly consistent with the primary findings, suggesting that our conclusions are robust across different age specifications. This supports the validity of our approach while preserving the advantages of using a broad population-based cohort.

### Comparison with prior work

Our findings suggested that long-term exposure to SO_2_, CO, PM_10_, PM_2.5_, NO_X_, NO, NO_2_, THC, NMHC, and CH_4_ acted consistently as a risk factor for higher incidence of infertility. Previous literature had discovered significant associations between increased levels of traffic-related air pollution, particularly the coarse fraction of particulate matter, and reduced fertility rates, with chronic exposure being more influential in infertility risk compared to short-term exposure [14]. The same trends were indicated in the infertility risk, with the pollutants of SO_2_, NO_2_. In a polluted area, Legro et al. found variable, cycle-dependent effects of declining air quality on reproductive outcomes after in vitro fertilization, with consistent associations between increased NO_2_ and lower live birth rates [15]. Furthermore, there is a suggestion that the likelihood of conception at the first unprotected menstrual cycle may decrease for couples exposed to average SO_2_ levels exceeding 40 μg/m^3^ during the second month before conception [16]. In comparison to Western countries, the PM₂.₅ concentrations observed in our study are relatively high. The average annual PM₂.₅ concentration in Taiwan was reported as 20.2 μg/m^3^ in 2023, which surpasses the WHO's recommended limit of 5 μg/m^3^. In contrast, the average PM₂.₅ concentrations in 2015 were 13.9 μg/m^3^ in Europe and 7–12 μg/m^3^ in the United States [17]. These elevated exposure levels in Taiwan highlight the potential public health burden and underscore the global relevance of our findings, especially for regions facing similar air pollution challenges.

The ovarian reserve is a crucial measure of a woman's reproductive potential, primarily determined by the quantity and quality of oocytes within the ovaries [18]. A noteworthy big-data approach by Santi et al. examined how environmental factors, particularly rising air pollution, impact anti-mullerian hormone (AMH) serum levels. While genetics play a major role in ovarian reserve at birth, this study suggests that environmental factors can also influence the decline in AMH and ovarian reserve during adulthood [19]. In contrast to our findings, Wu et al. [20] suggest that extended exposure to the air pollutant SO_2_ is linked to lower antral follicle count and a higher risk of poor ovarian response (< 4 oocytes retrieved), respectively, while NO_2_, PM_10_, PM_2.5_, CO, and O_3_ show no significant associations. These findings raise concerns about the potential adverse impact of atmospheric SO_2_ exposure on women's ovarian reserve.

O_3_, a secondary air pollutant formed from the combination of hydrocarbons and nitrogen oxides in sunlight, exhibits potential non-linearities in this process [21]. Legro et al. discovered a connection between O_3_ concentration at a patient's address and the likelihood of a live birth [15]. However, when accounting for NO_2_ and in vitro fertilization laboratory interactions, this association became insignificant. Moreover, Boulet et al. observed a weak positive correlation between O_3_ and implantation or live birth rates. Wu et al. identified higher O_3_ exposure in women with normal ovarian reserve (NOR) compared to poor responders [20]. However, multipollutant models showed no significant correlations.

### Possible mechanism

While the precise mechanisms behind air pollution-related female infertility are not yet fully understood, we propose a potential mechanism: maternal exposure to contaminants during the pre- and peri-conceptional periods could lead to abnormalities in oocytes, embryos, and fetuses [22]. This could potentially hinder safe childbirth, impact overall health and mental well-being, and thereby increase vulnerability to and susceptibility to infertility. Air pollutants disrupt both animal and human gametogenesis, reducing reproductive efficacy [8]. These endocrine-disrupting properties could activate the Ras/Erk pathway via interactions with nuclear receptors like estrogen or androgen receptors and specific cytosolic targets, while diesel exhaust particles and PM_2.5_ impact ovarian function by disrupting the endocrine system, increasing oxidative stress and inflammation, and activating specific targets that trigger abnormal MAPK signaling [23]. Indeed, in a recent study of 777 men, increased air pollutant concentrations were significantly associated with both the hypomethylation of F3, ICAM-1, and TLR-2, and the hypermethylation of IFN-γ and IL-6 [24]. What's most notable is that research has demonstrated that adding antioxidant factors to ovarian stimulation can enhance reproductive outcomes in women with polycystic ovarian syndrome [25].

Regarding ovarian function, a study did find a significant negative association between SO_2_ exposure throughout the entire antral follicle development stage and AFC [26]. This suggests that during the follicular vascularization transition stage, SO_2_ might be expedited in reaching follicles and granulosa cells, potentially contributing to impaired follicle development, hastened follicle loss, or altered ovarian function. The ovarian toxicity of environmental contaminants, characterized by meiotic disruption in oocyte formation resulting from genetic or epigenetic aberrations, can affect dormant follicles within the primordial pool that undergo growth stimulation and have the potential to develop into the antral stage [27]. Subsequently, these antral candidates are typically chosen to progress to the pre-ovulatory stage, marked by the transition from primary to pre-antral, during which the oocyte within the follicle enlarges, granulosa cells proliferate, and a layer of theca cells forms outside the granulosa cells, serving as a conduit for transporting steroids and growth factors to the granulosa cells to promote follicle vascularization [28]. Additionally, this bloodstream enables toxins to reach follicles, where they can suppress granulosa cell proliferation and growth. Furthermore, SO_2_ toxicology in mammalian organ development involves cellular injuries caused by inflammation and oxidative stress, such as lipid peroxidation, DNA damage, protein oxidation, and interference with signal transduction [29].

While ozone (O₃) is traditionally considered a harmful air pollutant, emerging evidence suggests that under certain controlled clinical settings with specific dosing protocols, ozone therapy may exert anti-inflammatory and antioxidant effects. For instance, medical ozone therapy administered in controlled healthcare environments has shown potential benefits in conditions such as HIV/AIDS, fibromyalgia, and peripheral arterial disease [30–32]. Some studies have explored its use in reproductive medicine, including pelvic inflammatory disease and tubal infertility [31]. It is crucial to emphasize that these therapeutic effects are achieved through controlled medical administration and are not equivalent to ambient environmental ozone exposure, which remains harmful to human health. However, the negative association observed in our study between ozone exposure and infertility risk is counterintuitive and must be interpreted with caution. Ambient ozone levels are influenced by seasonal and geographic factors and may act as a proxy for lower traffic-related co-pollutants. Additionally, negative confounding or interaction with other pollutants cannot be ruled out. Further research is needed to clarify this unexpected finding. This negative trend may reflect negative confounding, as ozone concentrations are often higher in suburban areas with lower levels of traffic-related pollutants. It is also possible that ecological bias affected the results, given that area-level monitoring data may not precisely represent individual-level exposure. These findings highlight the need for future studies to incorporate more detailed exposure metrics and consider pollutant interactions to better understand the role of ozone in reproductive health.

This study possesses several notable strengths. Firstly, it is the inaugural investigation to reveal the association between long-term exposure to air pollution and female infertility. Secondly, our analysis encompassed a comprehensive range of pollutants and utilized a significantly large sample, bolstering the statistical robustness of the findings. Thirdly, we conducted stratified analyses considering potential confounding factors such as age, urbanization level, insurance amount, CCI score, ambient temperature, season, and lag0-2, along with a wide spectrum of demographic characteristics. Furthermore, in registry-based studies, a minimum follow-up threshold is often necessary to ensure valid exposure-outcome relationships. Our choice of a 5-year minimum follow-up reflects this principle and strengthens the internal validity of the analysis. While it may reduce inclusion of short-term registrants, the large sample size and nationwide coverage help preserve overall representativeness. Lastly, the population-based data used in this study is representative of the general population in Taiwan.

This study has several limitations. First, key reproductive and lifestyle factors such as smoking status, alcohol consumption, body mass index (BMI), marital status, and oral contraceptive use were not available in the NHIRD. Although proxy variables—such as comorbidities, insurance level, and urbanization—were included in the adjusted models, the potential for residual confounding remains. Second, clinical variables such as disease severity, laboratory data, and symptomatology were unavailable, limiting the granularity of outcome characterization. Third, both exposure and outcome misclassification are possible. Exposure was estimated based on insurance-registered residential postal codes, which may not reflect actual pollutant exposure due to mobility or address reporting errors. Although we incorporated updated residential records to reduce spatial misclassification, some inaccuracy may persist. Outcome misclassification may also occur, particularly in defining infertility, as the database does not capture fertility intention, menopausal status, male partner fertility status, or utilization of assisted reproductive technologies—all of which may influence diagnostic classification. Additionally, death was inferred from withdrawal from the National Health Insurance program, which may not be completely accurate. Fourth, the retrospective nature of the study limits causal inference, and unmeasured or unknown confounders cannot be excluded. Finally, while the use of a five-year follow-up period strengthens the exposure-outcome temporality, it may inadvertently exclude short-term insurance registrants, potentially introducing selection bias. Nonetheless, the large sample size and nationwide coverage enhance the generalizability of the findings. Despite these limitations, the results provide consistent epidemiologic evidence supporting the association between long-term exposure to SO₂, CO, PM₁₀, PM₂.₅, NOₓ, NO, NO₂, THC, NMHC, and CH₄ and increased risk of female infertility.

## Conclusion

This study illustrates the considerable influence of air pollution on female infertility, revealing a distinct dose–response correlation between exposure to different pollutants and infertility rates. Prolonged exposure to SO2, CO, PM10, PM2.5, NOX, NO, NO2, THC, NMHC, and CH4 heightened infertility risks, while ozone exposure mitigated them. Our results underscore the importance of heightened awareness among clinicians and government stakeholders. In particular, targeted urban planning and environmental policies aimed at reducing NO₂ and PM exposure among women of reproductive age may serve as crucial public health interventions. They emphasize the necessity for extensive initiatives to curb air pollution and its impact on reproductive health. Further investigation is warranted to elucidate the underlying mechanisms and guide public health policies.

## Supplementary Information

Supplementary Material 1.

## Data Availability

Data is provided within the manuscript or supplementary information files.

## Abbreviations

SO₂: Sulfur dioxide
CO: Carbon monoxide
O₃: Ozone
PM₁₀: Particulate matter < 10 μm in diameter
PM₂.₅: Particulate matter < 2.5 μm in diameter
NOₓ: Nitrogen oxides
NO: Nitrogen monoxide
NO₂: Nitrogen dioxide
THC: Total hydrocarbons
NMHC: Nonmethane hydrocarbons
CH₄: Methane
NHIRD: National Health Insurance Research Database
IRB: Institutional Review Board
CCI: Charlson Comorbidity Index
ICD-9-CM: International Classification of Diseases, 9th Revision, Clinical Modification
AFC: Antral follicle count
AMH: Anti-Müllerian hormone
MAPK: Mitogen-activated protein kinase
IFN-γ: Interferon gamma
IL-6: Interleukin 6
EPA: Environmental Protection Administration
HR: Hazard ratio
SD: Standard deviation
BMI: Body mass index
