# Supplementary material for: Long-term exposure to ambient air pollutants and female infertility risk: a population-based cohort study in Taiwan
Source: BMC Public Health. 2025 Sep 30;25:3162. doi: 10.1186/s12889-025-24213-x (PMC12487008; doi:10.1186/s12889-025-24213-x)
Supplement: Supplementary file 1 — Supplementary Material 1. [file 12889_2025_24213_MOESM1_ESM.docx]

**Supplementary**

Table S1-1. Characteristics of the study population among tertiles of SO_2_ exposure.

Table S1-2. Characteristics of the study population among tertiles of CO exposure.

Table S1-3. Characteristics of the study population among tertiles of O_3_ exposure.

Table S1-4. Characteristics of the study population among tertiles of PM_10_ exposure.

Table S1-5. Characteristics of the study population among tertiles of PM_2.5_ exposure.

Table S1-6. Characteristics of the study population among tertiles of NO_X_ exposure.

Table S1-7. Characteristics of the study population among tertiles of NO exposure.

Table S1-8. Characteristics of the study population among tertiles of NO_2_ exposure.

Table S1-9. Characteristics of the study population among tertiles of THC exposure.

Table S1-10. Characteristics of the study population among tertiles of NMHC exposure.

Table S1-11. Characteristics of the study population among tertiles of CH_4_ exposure.

Table S2. Pearson’s correlation analysis for air pollutants over the exposure period

Table S3. Hazard ratios of long-term O3 exposure at a 1.73-ppb increment associated with the incidence of Infertility

Table S4. Hazard ratios for incidence of infertility among women aged 15–45 years of long-term exposure at an SD increment.

Table S5. The dose–response association between air pollutants and the risk of Infertility among women aged 15–45 years

**Table S1-1. Characteristics of the study population among tertiles of SO2 exposure**

| **Characteristic** | **Tertiles of average daily SO2, n (%)** | | | **P value** | **Total (N = 232125)** |
| --- | --- | --- | --- | --- | --- |
|  | **T1 (lowest) (n = 77141)** | **T2 (n = 75237)** | **T3 (highest) (n = 79747)** |  |  |
| **Infertility** | 974 (1.26) | 1799 (2.39) | 2208 (2.77) | <0.001 | 4981 (2.15) |
| **Age, years** |  |  |  | <0.001 |  |
| 15-35 | 40365 (52.33) | 38943 (51.76) | 42424 (53.20) |  | 121732 (52.44) |
| 36-60 | 36776 (47.67) | 36294 (48.24) | 37323 (46.80) |  | 110393 (47.56) |
| Mean ± SD | 35.42 ± 12.21 | 34.88 ± 12.84 | 34.94 ± 12.31 | <0.001 | 35.08 ± 12.45 |
| **Urbanization level** |  |  |  | <0.001 |  |
| 1 (highest) | 45942 (59.56) | 49038 (65.18) | 43901 (55.05) |  | 138881 (59.83) |
| 2 | 25735 (33.36) | 18781 (24.96) | 26640 (33.41) |  | 71156 (30.65) |
| 3 | 3405 (4.41) | 5073 (6.74) | 5802 (7.28) |  | 14280 (6.15) |
| 4 (lowest) | 119 (0.15) | 492 (0.65) | 1215 (1.52) |  | 1826 (0.79) |
| unknown | 1940 (2.51) | 1853 (2.46) | 2189 (2.74) |  | 5982 (2.58) |
| **Insurance amount, NT$** |  |  |  | <0.001 |  |
| financially dependent | 583 (0.76) | 272 (0.36) | 488 (0.61) |  | 1343 (0.58) |
| 1-19999 | 23577 (30.56) | 26236 (34.87) | 30055 (37.69) |  | 79868 (34.41) |
| 20000-39999 | 34093 (44.20) | 34021 (45.22) | 34761 (43.59) |  | 102875 (44.32) |
| ≥40000 | 16547 (21.45) | 12664 (16.83) | 11816 (14.82) |  | 41027 (17.67) |
| unknown | 2341 (3.03) | 2044 (2.72) | 2627 (3.29) |  | 7012 (3.02) |
| **CCI score** |  |  |  | <0.001 |  |
| Mean ± SD | 1.10 ± 1.66 | 1.12 ± 1.70 | 1.07 ± 1.68 |  | 1.10 ± 1.68 |
| **Comorbidity** |  |  |  |  |  |
| Inflammatory disease of the ovary, fallopian tube, pelvic cellular tissue, and peritoneum | 10808 (14.01) | 11130 (14.79) | 13988 (17.54) | <0.001 | 35926 (15.48) |
| Inflammatory disease of the uterus | 3826 (4.96) | 4293 (5.71) | 4122 (5.17) | <0.001 | 12241 (5.27) |
| Inflammatory disease of the cervix, vagina, and vulva | 35231 (45.67) | 35205 (46.79) | 37367 (46.86) | <0.001 | 107803 (46.44) |
| Endometriosis | 2952 (3.83) | 2775 (3.69) | 3101 (3.89) | 0.110 | 8828 (3.80) |
| Hypertension | 12944 (16.78) | 12605 (16.75) | 13216 (16.57) | 0.486 | 38765 (16.70) |
| Diabetes mellitus | 9055 (11.74) | 9075 (12.06) | 9195 (11.53) | 0.005 | 27325 (11.77) |
| Hypertriglyceridemia | 106 (0.14) | 79 (0.10) | 101 (0.13) | 0.186 | 286 (0.12) |
| Hypercholesterolemia | 10023 (12.99) | 10266 (13.64) | 9681 (12.14) | <0.001 | 29970 (12.91) |
| Coronary artery disease | 7647 (9.91) | 7234 (9.61) | 6908 (8.66) | <0.001 | 21789 (9.39) |
| Disorders of eating | 85 (0.11) | 108 (0.14) | 109 (0.14) | 0.160 | 302 (0.13) |

SD, standard deviation; CCI score, Charlson Comorbidity Index score.
The tertile values, in ppb, were as follows: T1: < 3.59; T2: ≥ 3.59 and < 4.04; T3: ≥ 4.04.
The chi-squared test or one-way analysis of variance among tertiles of sulfur dioxide.
Urbanization level was defined at the beginning of the follow-up period.
Insurance amount was measured as the average value during the period of air pollutant exposure assessment.
Comorbidities were defined before the survival date.

**Table S1-2. Characteristics of the study population among tertiles of CO exposure**

| **Characteristic** | **Tertiles of average daily CO, n (%)** | | | **P value** | **Total (N = 232125)** |
| --- | --- | --- | --- | --- | --- |
|  | **T1 (lowest) (n = 77375)** | **T2 (n = 77375)** | **T3 (highest) (n = 77375)** |  |  |
| **Infertility** | 443 (0.57) | 1351 (1.75) | 3187 (4.12) | <0.001 | 4981 (2.15) |
| **Age, years** |  |  |  | <0.001 |  |
| 15-35 | 35622 (46.04) | 42545 (54.99) | 43565 (56.30) |  | 121732 (52.44) |
| 36-60 | 41753 (53.96) | 34830 (45.01) | 33810 (43.70) |  | 110393 (47.56) |
| Mean ± SD | 36.74 ± 12.53 | 34.30 ± 12.59 | 34.19 ± 12.07 | <0.001 | 35.08 ± 12.45 |
| **Urbanization level** |  |  |  | <0.001 |  |
| 1 (highest) | 44483 (57.49) | 44068 (56.95) | 50330 (65.05) |  | 138881 (59.83) |
| 2 | 24683 (31.90) | 27441 (35.46) | 19032 (24.60) |  | 71156 (30.65) |
| 3 | 5456 (7.05) | 3683 (4.76) | 5141 (6.64) |  | 14280 (6.15) |
| 4 (lowest) | 217 (0.28) | 694 (0.90) | 915 (1.18) |  | 1826 (0.79) |
| unknown | 2536 (3.28) | 1489 (1.92) | 1957 (2.53) |  | 5982 (2.58) |
| **Insurance amount, NT$** |  |  |  | <0.001 |  |
| financially dependent | 526 (0.68) | 358 (0.46) | 459 (0.59) |  | 1343 (0.58) |
| 1-19999 | 25360 (32.78) | 29569 (38.22) | 24939 (32.23) |  | 79868 (34.41) |
| 20000-39999 | 37492 (48.45) | 33283 (43.02) | 32100 (41.49) |  | 102875 (44.32) |
| ≥40000 | 11688 (15.11) | 11780 (15.22) | 17559 (22.69) |  | 41027 (17.67) |
| unknown | 2309 (2.98) | 2385 (3.08) | 2318 (3.00) |  | 7012 (3.02) |
| **CCI score** |  |  |  | <0.001 |  |
| Mean ± SD | 1.22 ± 1.75 | 1.05 ± 1.65 | 1.02 ± 1.64 |  | 1.10 ± 1.68 |
| **Comorbidity** |  |  |  |  |  |
| Inflammatory disease of the ovary, fallopian tube, pelvic cellular tissue, and peritoneum | 13350 (17.25) | 11930 (15.42) | 10646 (13.76) | <0.001 | 35926 (15.48) |
| Inflammatory disease of the uterus | 3834 (4.96) | 3688 (4.77) | 4719 (6.10) | <0.001 | 12241 (5.27) |
| Inflammatory disease of the cervix, vagina, and vulva | 36392 (47.03) | 35608 (46.02) | 35803 (46.27) | <0.001 | 107803 (46.44) |
| Endometriosis | 2762 (3.57) | 2926 (3.78) | 3140 (4.06) | <0.001 | 8828 (3.80) |
| Hypertension | 15178 (19.62) | 12237 (15.82) | 11350 (14.67) | <0.001 | 38765 (16.70) |
| Diabetes mellitus | 10733 (13.87) | 8619 (11.14) | 7973 (10.30) | <0.001 | 27325 (11.77) |
| Hypertriglyceridemia | 117 (0.15) | 97 (0.13) | 72 (0.09) | 0.005 | 286 (0.12) |
| Hypercholesterolemia | 11086 (14.33) | 9078 (11.73) | 9806 (12.67) | <0.001 | 29970 (12.91) |
| Coronary artery disease | 8881 (11.48) | 6613 (8.55) | 6295 (8.14) | <0.001 | 21789 (9.39) |
| Disorders of eating | 96 (0.12) | 98 (0.13) | 108 (0.14) | 0.663 | 302 (0.13) |

SD, standard deviation; CCI score, Charlson Comorbidity Index score.
The tertile values, in ppm, were as follows: T1: < 0.48; T2: ≥ 0.48 and < 0.59; T3: ≥ 0.59.
The chi-squared test or one-way analysis of variance among tertiles of carbon monoxide.
Urbanization level was defined at the beginning of the follow-up period.
Insurance amount was measured as the average value during the period of air pollutant exposure assessment.
Comorbidities were defined before the survival date.

**Table S1-3. Characteristics of the study population among tertiles of O3 exposure**

| **Characteristic** | **Tertiles of average daily O3, n (%)** | | | **P value** | **Total (N = 232125)** |
| --- | --- | --- | --- | --- | --- |
|  | **T1 (lowest) (n = 77372)** | **T2 (n = 77365)** | **T3 (highest) (n = 77388)** |  |  |
| **Infertility** | 3579 (4.63) | 796 (1.03) | 606 (0.78) | <0.001 | 4981 (2.15) |
| **Age, years** |  |  |  | <0.001 |  |
| 15-35 | 43942 (56.79) | 42758 (55.27) | 35032 (45.27) |  | 121732 (52.44) |
| 36-60 | 33430 (43.21) | 34607 (44.73) | 42356 (54.73) |  | 110393 (47.56) |
| Mean ± SD | 34.06 ± 12.08 | 34.34 ± 12.50 | 36.83 ± 12.59 | <0.001 | 35.08 ± 12.45 |
| **Urbanization level** |  |  |  | <0.001 |  |
| 1 (highest) | 50915 (65.81) | 44176 (57.10) | 43790 (56.58) |  | 138881 (59.83) |
| 2 | 19998 (25.85) | 26042 (33.66) | 25116 (32.45) |  | 71156 (30.65) |
| 3 | 3897 (5.04) | 5092 (6.58) | 5291 (6.84) |  | 14280 (6.15) |
| 4 (lowest) | 672 (0.87) | 428 (0.55) | 726 (0.94) |  | 1826 (0.79) |
| unknown | 1890 (2.44) | 1627 (2.10) | 2465 (3.19) |  | 5982 (2.58) |
| **Insurance amount, NT$** |  |  |  | <0.001 |  |
| financially dependent | 506 (0.65) | 360 (0.47) | 477 (0.62) |  | 1343 (0.58) |
| 1-19999 | 23985 (31.00) | 29866 (38.60) | 26017 (33.62) |  | 79868 (34.41) |
| 20000-39999 | 31844 (41.16) | 34214 (44.22) | 36817 (47.57) |  | 102875 (44.32) |
| ≥40000 | 18699 (24.17) | 10689 (13.82) | 11639 (15.04) |  | 41027 (17.67) |
| unknown | 2338 (3.02) | 2236 (2.89) | 2438 (3.15) |  | 7012 (3.02) |
| **CCI score** |  |  |  | <0.001 |  |
| Mean ± SD | 1.05 ± 1.68 | 1.03 ± 1.61 | 1.22 ± 1.75 |  | 1.10 ± 1.68 |
| **Comorbidity** |  |  |  |  |  |
| Inflammatory disease of the ovary, fallopian tube, pelvic cellular tissue, and peritoneum | 10376 (13.41) | 11893 (15.37) | 13657 (17.65) | <0.001 | 35926 (15.48) |
| Inflammatory disease of the uterus | 4459 (5.76) | 3934 (5.08) | 3848 (4.97) | <0.001 | 12241 (5.27) |
| Inflammatory disease of the cervix, vagina, and vulva | 35754 (46.21) | 36004 (46.54) | 36045 (46.58) | 0.284 | 107803 (46.44) |
| Endometriosis | 3121 (4.03) | 2791 (3.61) | 2916 (3.77) | <0.001 | 8828 (3.80) |
| Hypertension | 11241 (14.53) | 12264 (15.85) | 15260 (19.72) | <0.001 | 38765 (16.70) |
| Diabetes mellitus | 7997 (10.34) | 8529 (11.02) | 10799 (13.95) | <0.001 | 27325 (11.77) |
| Hypertriglyceridemia | 75 (0.10) | 101 (0.13) | 110 (0.14) | 0.031 | 286 (0.12) |
| Hypercholesterolemia | 9617 (12.43) | 9044 (11.69) | 11309 (14.61) | <0.001 | 29970 (12.91) |
| Coronary artery disease | 6365 (8.23) | 6793 (8.78) | 8631 (11.15) | <0.001 | 21789 (9.39) |
| Disorders of eating | 95 (0.12) | 100 (0.13) | 107 (0.14) | 0.698 | 302 (0.13) |

SD, standard deviation; CCI score, Charlson Comorbidity Index score.
The tertile values, in ppb, were as follows: T1: < 27.77; T2: ≥ 27.77 and < 29.11; T3: ≥ 29.11.
The chi-squared test or one-way analysis of variance among tertiles of ozone.
Urbanization level was defined at the beginning of the follow-up period.
Insurance amount was measured as the average value during the period of air pollutant exposure assessment.
Comorbidities were defined before the survival date.

**Table S1-4. Characteristics of the study population among tertiles of PM10 exposure**

| **Characteristic** | **Tertiles of average daily PM10, n (%)** | | | **P value** | **Total (N = 232125)** |
| --- | --- | --- | --- | --- | --- |
|  | **T1 (lowest) (n = 77360)** | **T2 (n = 72522)** | **T3 (highest) (n = 82243)** |  |  |
| **Infertility** | 1243 (1.61) | 1681 (2.32) | 2057 (2.50) | <0.001 | 4981 (2.15) |
| **Age, years** |  |  |  | <0.001 |  |
| 15-35 | 42279 (54.65) | 37015 (51.04) | 42438 (51.60) |  | 121732 (52.44) |
| 36-60 | 35081 (45.35) | 35507 (48.96) | 39805 (48.40) |  | 110393 (47.56) |
| Mean ± SD | 34.64 ± 12.00 | 35.12 ± 12.89 | 35.46 ± 12.46 | <0.001 | 35.08 ± 12.45 |
| **Urbanization level** |  |  |  | <0.001 |  |
| 1 (highest) | 52730 (68.16) | 44124 (60.84) | 42027 (51.10) |  | 138881 (59.83) |
| 2 | 17533 (22.66) | 21382 (29.48) | 32241 (39.20) |  | 71156 (30.65) |
| 3 | 4488 (5.80) | 4654 (6.42) | 5138 (6.25) |  | 14280 (6.15) |
| 4 (lowest) | 767 (0.99) | 375 (0.52) | 684 (0.83) |  | 1826 (0.79) |
| unknown | 1842 (2.38) | 1987 (2.74) | 2153 (2.62) |  | 5982 (2.58) |
| **Insurance amount, NT$** |  |  |  | <0.001 |  |
| financially dependent | 518 (0.67) | 283 (0.39) | 542 (0.66) |  | 1343 (0.58) |
| 1-19999 | 23042 (29.79) | 25477 (35.13) | 31349 (38.12) |  | 79868 (34.41) |
| 20000-39999 | 33075 (42.75) | 32659 (45.03) | 37141 (45.16) |  | 102875 (44.32) |
| ≥40000 | 18714 (24.19) | 11998 (16.54) | 10315 (12.54) |  | 41027 (17.67) |
| unknown | 2011 (2.60) | 2105 (2.90) | 2896 (3.52) |  | 7012 (3.02) |
| **CCI score** |  |  |  | <0.001 |  |
| Mean ± SD | 1.02 ± 1.58 | 1.11 ± 1.70 | 1.16 ± 1.75 |  | 1.10 ± 1.68 |
| **Comorbidity** |  |  |  |  |  |
| Inflammatory disease of the ovary, fallopian tube, pelvic cellular tissue, and peritoneum | 10471 (13.54) | 10635 (14.66) | 14820 (18.02) | <0.001 | 35926 (15.48) |
| Inflammatory disease of the uterus | 4840 (6.26) | 3567 (4.92) | 3834 (4.66) | <0.001 | 12241 (5.27) |
| Inflammatory disease of the cervix, vagina, and vulva | 36151 (46.73) | 33033 (45.55) | 38619 (46.96) | <0.001 | 107803 (46.44) |
| Endometriosis | 3017 (3.90) | 2425 (3.34) | 3386 (4.12) | <0.001 | 8828 (3.80) |
| Hypertension | 12126 (15.67) | 12464 (17.19) | 14175 (17.24) | <0.001 | 38765 (16.70) |
| Diabetes mellitus | 8395 (10.85) | 8775 (12.10) | 10155 (12.35) | <0.001 | 27325 (11.77) |
| Hypertriglyceridemia | 68 (0.09) | 87 (0.12) | 131 (0.16) | <0.001 | 286 (0.12) |
| Hypercholesterolemia | 10567 (13.66) | 9875 (13.62) | 9528 (11.59) | <0.001 | 29970 (12.91) |
| Coronary artery disease | 6785 (8.77) | 7079 (9.76) | 7925 (9.64) | <0.001 | 21789 (9.39) |
| Disorders of eating | 97 (0.13) | 88 (0.12) | 117 (0.14) | 0.473 | 302 (0.13) |

SD, standard deviation; CCI score, Charlson Comorbidity Index score.
The tertile values, in μg/m3, were as follows: T1: < 47.66; T2: ≥ 47.66 and < 56.18; T3: ≥ 56.18.
The chi-squared test or one-way analysis of variance among tertiles of particulate matter <10 μm in aerodynamic diameter.
Urbanization level was defined at the beginning of the follow-up period.
Insurance amount was measured as the average value during the period of air pollutant exposure assessment.
Comorbidities were defined before the survival date.

**Table S1-5. Characteristics of the study population among tertiles of PM2.5 exposure**

| **Characteristic** | **Tertiles of average daily PM2.5, n (%)** | | | **P value** | **Total (N = 232108)** |
| --- | --- | --- | --- | --- | --- |
|  | **T1 (lowest) (n = 77369)** | **T2 (n = 75510)** | **T3 (highest) (n = 79229)** |  |  |
| **Infertility** | 832 (1.08) | 2077 (2.75) | 2064 (2.61) | <0.001 | 4973 (2.14) |
| **Age, years** |  |  |  | <0.001 |  |
| 15-35 | 42347 (54.73) | 39321 (52.07) | 40053 (50.55) |  | 121721 (52.44) |
| 36-60 | 35022 (45.27) | 36189 (47.93) | 39176 (49.45) |  | 110387 (47.56) |
| Mean ± SD | 34.60 ± 11.92 | 34.82 ± 12.92 | 35.79 ± 12.48 | <0.001 | 35.08 ± 12.45 |
| **Urbanization level** |  |  |  | <0.001 |  |
| 1 (highest) | 51416 (66.46) | 46672 (61.81) | 40784 (51.48) |  | 138872 (59.83) |
| 2 | 18860 (24.38) | 21946 (29.06) | 30344 (38.30) |  | 71150 (30.65) |
| 3 | 4421 (5.71) | 4625 (6.13) | 5232 (6.60) |  | 14278 (6.15) |
| 4 (lowest) | 770 (1.00) | 381 (0.50) | 675 (0.85) |  | 1826 (0.79) |
| unknown | 1902 (2.46) | 1886 (2.50) | 2194 (2.77) |  | 5982 (2.58) |
| **Insurance amount, NT$** |  |  |  | <0.001 |  |
| financially dependent | 531 (0.69) | 274 (0.36) | 538 (0.68) |  | 1343 (0.58) |
| 1-19999 | 24000 (31.02) | 26203 (34.70) | 29655 (37.43) |  | 79858 (34.41) |
| 20000-39999 | 32677 (42.24) | 33785 (44.74) | 36410 (45.96) |  | 102872 (44.32) |
| ≥40000 | 18101 (23.40) | 13090 (17.34) | 9834 (12.41) |  | 41025 (17.67) |
| unknown | 2060 (2.66) | 2158 (2.86) | 2792 (3.52) |  | 7010 (3.02) |
| **CCI score** |  |  |  | <0.001 |  |
| Mean ± SD | 1.01 ± 1.56 | 1.10 ± 1.69 | 1.18 ± 1.78 |  | 1.10 ± 1.68 |
| **Comorbidity** |  |  |  |  |  |
| Inflammatory disease of the ovary, fallopian tube, pelvic cellular tissue, and peritoneum | 11040 (14.27) | 10639 (14.09) | 14247 (17.98) | <0.001 | 35926 (15.48) |
| Inflammatory disease of the uterus | 4693 (6.07) | 3833 (5.08) | 3713 (4.69) | <0.001 | 12239 (5.27) |
| Inflammatory disease of the cervix, vagina, and vulva | 36314 (46.94) | 34478 (45.66) | 37007 (46.71) | <0.001 | 107799 (46.44) |
| Endometriosis | 2936 (3.79) | 2688 (3.56) | 3204 (4.04) | <0.001 | 8828 (3.80) |
| Hypertension | 12048 (15.57) | 12759 (16.90) | 13955 (17.61) | <0.001 | 38762 (16.70) |
| Diabetes mellitus | 8331 (10.77) | 9011 (11.93) | 9979 (12.60) | <0.001 | 27321 (11.77) |
| Hypertriglyceridemia | 55 (0.07) | 99 (0.13) | 132 (0.17) | <0.001 | 286 (0.12) |
| Hypercholesterolemia | 10613 (13.72) | 9853 (13.05) | 9503 (11.99) | <0.001 | 29969 (12.91) |
| Coronary artery disease | 6482 (8.38) | 7322 (9.70) | 7982 (10.07) | <0.001 | 21786 (9.39) |
| Disorders of eating | 94 (0.12) | 97 (0.13) | 111 (0.14) | 0.587 | 302 (0.13) |

SD, standard deviation; CCI score, Charlson Comorbidity Index score.
The tertile values, in μg/m3, were as follows: T1: < 27.67; T2: ≥ 27.67 and < 34.95; T3: ≥ 34.95.
The chi-squared test or one-way analysis of variance among tertiles of particulate matter <2.5 μm in aerodynamic diameter.
Urbanization level was defined at the beginning of the follow-up period.
Insurance amount was measured as the average value during the period of air pollutant exposure assessment.
Comorbidities were defined before the survival date.

**Table S1-6. Characteristics of the study population among tertiles of NOX exposure**

| **Characteristic** | **Tertiles of average daily NOX, n (%)** | | | **P value** | **Total (N = 232125)** |
| --- | --- | --- | --- | --- | --- |
|  | **T1 (lowest) (n = 77370)** | **T2 (n = 77380)** | **T3 (highest) (n = 77375)** |  |  |
| **Infertility** | 603 (0.78) | 1778 (2.30) | 2600 (3.36) | <0.001 | 4981 (2.15) |
| **Age, years** |  |  |  | <0.001 |  |
| 15-35 | 34986 (45.22) | 43061 (55.65) | 43685 (56.46) |  | 121732 (52.44) |
| 36-60 | 42384 (54.78) | 34319 (44.35) | 33690 (43.54) |  | 110393 (47.56) |
| Mean ± SD | 36.96 ± 12.46 | 34.13 ± 12.64 | 34.14 ± 12.04 | <0.001 | 35.08 ± 12.45 |
| **Urbanization level** |  |  |  | <0.001 |  |
| 1 (highest) | 44856 (57.98) | 43582 (56.32) | 50443 (65.19) |  | 138881 (59.83) |
| 2 | 24451 (31.60) | 27768 (35.89) | 18937 (24.47) |  | 71156 (30.65) |
| 3 | 5294 (6.84) | 3836 (4.96) | 5150 (6.66) |  | 14280 (6.15) |
| 4 (lowest) | 189 (0.24) | 716 (0.93) | 921 (1.19) |  | 1826 (0.79) |
| unknown | 2580 (3.33) | 1478 (1.91) | 1924 (2.49) |  | 5982 (2.58) |
| **Insurance amount, NT$** |  |  |  | <0.001 |  |
| financially dependent | 540 (0.70) | 341 (0.44) | 462 (0.60) |  | 1343 (0.58) |
| 1-19999 | 25269 (32.66) | 29873 (38.61) | 24726 (31.96) |  | 79868 (34.41) |
| 20000-39999 | 37360 (48.29) | 33187 (42.89) | 32328 (41.78) |  | 102875 (44.32) |
| ≥40000 | 11876 (15.35) | 11566 (14.95) | 17585 (22.73) |  | 41027 (17.67) |
| unknown | 2325 (3.01) | 2413 (3.12) | 2274 (2.94) |  | 7012 (3.02) |
| **CCI score** |  |  |  | <0.001 |  |
| Mean ± SD | 1.25 ± 1.77 | 1.05 ± 1.66 | 0.99 ± 1.59 |  | 1.10 ± 1.68 |
| **Comorbidity** |  |  |  |  |  |
| Inflammatory disease of the ovary, fallopian tube, pelvic cellular tissue, and peritoneum | 13287 (17.17) | 11949 (15.44) | 10690 (13.82) | <0.001 | 35926 (15.48) |
| Inflammatory disease of the uterus | 3771 (4.87) | 3722 (4.81) | 4748 (6.14) | <0.001 | 12241 (5.27) |
| Inflammatory disease of the cervix, vagina, and vulva | 36381 (47.02) | 35503 (45.88) | 35919 (46.42) | <0.001 | 107803 (46.44) |
| Endometriosis | 2764 (3.57) | 2925 (3.78) | 3139 (4.06) | <0.001 | 8828 (3.80) |
| Hypertension | 15374 (19.87) | 12055 (15.58) | 11336 (14.65) | <0.001 | 38765 (16.70) |
| Diabetes mellitus | 10952 (14.16) | 8498 (10.98) | 7875 (10.18) | <0.001 | 27325 (11.77) |
| Hypertriglyceridemia | 116 (0.15) | 98 (0.13) | 72 (0.09) | 0.006 | 286 (0.12) |
| Hypercholesterolemia | 11263 (14.56) | 8916 (11.52) | 9791 (12.65) | <0.001 | 29970 (12.91) |
| Coronary artery disease | 9070 (11.72) | 6515 (8.42) | 6204 (8.02) | <0.001 | 21789 (9.39) |
| Disorders of eating | 91 (0.12) | 100 (0.13) | 111 (0.14) | 0.369 | 302 (0.13) |

SD, standard deviation; CCI score, Charlson Comorbidity Index score.
The tertile values, in ppb, were as follows: T1: < 21.44; T2: ≥ 21.44 and < 29.60; T3: ≥ 29.60.
The chi-squared test or one-way analysis of variance among tertiles of nitrogen oxides.
Urbanization level was defined at the beginning of the follow-up period.
Insurance amount was measured as the average value during the period of air pollutant exposure assessment.
Comorbidities were defined before the survival date.

**Table S1-7. Characteristics of the study population among tertiles of NO exposure**

| **Characteristic** | **Tertiles of average daily NO, n (%)** | | | **P value** | **Total (N = 232125)** |
| --- | --- | --- | --- | --- | --- |
|  | **T1 (lowest) (n = 77375)** | **T2 (n = 77374)** | **T3 (highest) (n = 77376)** |  |  |
| **Infertility** | 561 (0.73) | 1839 (2.38) | 2581 (3.34) | <0.001 | 4981 (2.15) |
| **Age, years** |  |  |  | <0.001 |  |
| 15-35 | 35749 (46.20) | 41842 (54.08) | 44141 (57.05) |  | 121732 (52.44) |
| 36-60 | 41626 (53.80) | 35532 (45.92) | 33235 (42.95) |  | 110393 (47.56) |
| Mean ± SD | 36.83 ± 12.42 | 34.51 ± 12.57 | 33.90 ± 12.18 | <0.001 | 35.08 ± 12.45 |
| **Urbanization level** |  |  |  | <0.001 |  |
| 1 (highest) | 42459 (54.87) | 44567 (57.60) | 51855 (67.02) |  | 138881 (59.83) |
| 2 | 26281 (33.97) | 27572 (35.63) | 17303 (22.36) |  | 71156 (30.65) |
| 3 | 5887 (7.61) | 2824 (3.65) | 5569 (7.20) |  | 14280 (6.15) |
| 4 (lowest) | 167 (0.22) | 685 (0.89) | 974 (1.26) |  | 1826 (0.79) |
| unknown | 2581 (3.34) | 1726 (2.23) | 1675 (2.16) |  | 5982 (2.58) |
| **Insurance amount, NT$** |  |  |  | <0.001 |  |
| financially dependent | 567 (0.73) | 383 (0.50) | 393 (0.51) |  | 1343 (0.58) |
| 1-19999 | 25682 (33.19) | 29423 (38.03) | 24763 (32.00) |  | 79868 (34.41) |
| 20000-39999 | 37531 (48.51) | 32508 (42.01) | 32836 (42.44) |  | 102875 (44.32) |
| ≥40000 | 11127 (14.38) | 12644 (16.34) | 17256 (22.30) |  | 41027 (17.67) |
| unknown | 2468 (3.19) | 2416 (3.12) | 2128 (2.75) |  | 7012 (3.02) |
| **CCI score** |  |  |  | <0.001 |  |
| Mean ± SD | 1.23 ± 1.76 | 1.08 ± 1.69 | 0.98 ± 1.58 |  | 1.10 ± 1.68 |
| **Comorbidity** |  |  |  |  |  |
| Inflammatory disease of the ovary, fallopian tube, pelvic cellular tissue, and peritoneum | 13241 (17.11) | 12540 (16.21) | 10145 (13.11) | <0.001 | 35926 (15.48) |
| Inflammatory disease of the uterus | 3548 (4.59) | 3860 (4.99) | 4833 (6.25) | <0.001 | 12241 (5.27) |
| Inflammatory disease of the cervix, vagina, and vulva | 36347 (46.98) | 35805 (46.28) | 35651 (46.08) | <0.001 | 107803 (46.44) |
| Endometriosis | 2742 (3.54) | 3035 (3.92) | 3051 (3.94) | <0.001 | 8828 (3.80) |
| Hypertension | 15120 (19.54) | 12413 (16.04) | 11232 (14.52) | <0.001 | 38765 (16.70) |
| Diabetes mellitus | 10708 (13.84) | 8831 (11.41) | 7786 (10.06) | <0.001 | 27325 (11.77) |
| Hypertriglyceridemia | 122 (0.16) | 99 (0.13) | 65 (0.08) | <0.001 | 286 (0.12) |
| Hypercholesterolemia | 10676 (13.80) | 9182 (11.87) | 10112 (13.07) | <0.001 | 29970 (12.91) |
| Coronary artery disease | 8928 (11.54) | 6553 (8.47) | 6308 (8.15) | <0.001 | 21789 (9.39) |
| Disorders of eating | 83 (0.11) | 112 (0.14) | 107 (0.14) | 0.092 | 302 (0.13) |

SD, standard deviation; CCI score, Charlson Comorbidity Index score.
The tertile values, in ppb, were as follows: T1: < 4.93; T2: ≥ 4.93 and < 8.51; T3: ≥ 8.51.
The chi-squared test or one-way analysis of variance among tertiles of nitric oxide.
Urbanization level was defined at the beginning of the follow-up period.
Insurance amount was measured as the average value during the period of air pollutant exposure assessment.
Comorbidities were defined before the survival date.

**Table S1-8. Characteristics of the study population among tertiles of NO2 exposure**

| **Characteristic** | **Tertiles of average daily NO2, n (%)** | | | **P value** | **Total (N = 232125)** |
| --- | --- | --- | --- | --- | --- |
|  | **T1 (lowest) (n = 77348)** | **T2 (n = 77402)** | **T3 (highest) (n = 77375)** |  |  |
| **Infertility** | 675 (0.87) | 1506 (1.95) | 2800 (3.62) | <0.001 | 4981 (2.15) |
| **Age, years** |  |  |  | <0.001 |  |
| 15-35 | 35143 (45.43) | 42781 (55.27) | 43808 (56.62) |  | 121732 (52.44) |
| 36-60 | 42205 (54.57) | 34621 (44.73) | 33567 (43.38) |  | 110393 (47.56) |
| Mean ± SD | 36.86 ± 12.60 | 34.26 ± 12.52 | 34.11 ± 12.04 | <0.001 | 35.08 ± 12.45 |
| **Urbanization level** |  |  |  | <0.001 |  |
| 1 (highest) | 44654 (57.73) | 44243 (57.16) | 49984 (64.60) |  | 138881 (59.83) |
| 2 | 24483 (31.65) | 26882 (34.73) | 19791 (25.58) |  | 71156 (30.65) |
| 3 | 5530 (7.15) | 3970 (5.13) | 4780 (6.18) |  | 14280 (6.15) |
| 4 (lowest) | 231 (0.30) | 715 (0.92) | 880 (1.14) |  | 1826 (0.79) |
| unknown | 2450 (3.17) | 1592 (2.06) | 1940 (2.51) |  | 5982 (2.58) |
| **Insurance amount, NT$** |  |  |  | <0.001 |  |
| financially dependent | 518 (0.67) | 365 (0.47) | 460 (0.59) |  | 1343 (0.58) |
| 1-19999 | 25431 (32.88) | 29321 (37.88) | 25116 (32.46) |  | 79868 (34.41) |
| 20000-39999 | 37288 (48.21) | 33435 (43.20) | 32152 (41.55) |  | 102875 (44.32) |
| ≥40000 | 11864 (15.34) | 11791 (15.23) | 17372 (22.45) |  | 41027 (17.67) |
| unknown | 2247 (2.91) | 2490 (3.22) | 2275 (2.94) |  | 7012 (3.02) |
| **CCI score** |  |  |  | <0.001 |  |
| Mean ± SD | 1.24 ± 1.78 | 1.05 ± 1.64 | 1.00 ± 1.61 |  | 1.10 ± 1.68 |
| **Comorbidity** |  |  |  |  |  |
| Inflammatory disease of the ovary, fallopian tube, pelvic cellular tissue, and peritoneum | 12929 (16.72) | 12025 (15.54) | 10972 (14.18) | <0.001 | 35926 (15.48) |
| Inflammatory disease of the uterus | 3858 (4.99) | 3719 (4.80) | 4664 (6.03) | <0.001 | 12241 (5.27) |
| Inflammatory disease of the cervix, vagina, and vulva | 36192 (46.79) | 35654 (46.06) | 35957 (46.47) | 0.016 | 107803 (46.44) |
| Endometriosis | 2663 (3.44) | 2978 (3.85) | 3187 (4.12) | <0.001 | 8828 (3.80) |
| Hypertension | 15355 (19.85) | 12164 (15.72) | 11246 (14.53) | <0.001 | 38765 (16.70) |
| Diabetes mellitus | 10878 (14.06) | 8563 (11.06) | 7884 (10.19) | <0.001 | 27325 (11.77) |
| Hypertriglyceridemia | 112 (0.14) | 104 (0.13) | 70 (0.09) | 0.005 | 286 (0.12) |
| Hypercholesterolemia | 11304 (14.61) | 9077 (11.73) | 9589 (12.39) | <0.001 | 29970 (12.91) |
| Coronary artery disease | 9100 (11.77) | 6517 (8.42) | 6172 (7.98) | <0.001 | 21789 (9.39) |
| Disorders of eating | 92 (0.12) | 95 (0.12) | 115 (0.15) | 0.211 | 302 (0.13) |

SD, standard deviation; CCI score, Charlson Comorbidity Index score.
The tertile values, in ppb, were as follows: T1: < 16.55; T2: ≥ 16.55 and < 20.49; T3: ≥ 20.49.
The chi-squared test or one-way analysis of variance among tertiles of nitrogen dioxide.
Urbanization level was defined at the beginning of the follow-up period.
Insurance amount was measured as the average value during the period of air pollutant exposure assessment.
Comorbidities were defined before the survival date.

**Table S1-9. Characteristics of the study population among tertiles of THC exposure**

| **Characteristic** | **Tertiles of average daily THC, n (%)** | | | **P value** | **Total (N = 230449)** |
| --- | --- | --- | --- | --- | --- |
|  | **T1 (lowest) (n = 76816)** | **T2 (n = 76816)** | **T3 (highest) (n = 76817)** |  |  |
| **Infertility** | 268 (0.35) | 1084 (1.41) | 3608 (4.70) | <0.001 | 4960 (2.15) |
| **Age, years** |  |  |  | <0.001 |  |
| 15-35 | 37769 (49.17) | 40631 (52.89) | 42444 (55.25) |  | 120844 (52.44) |
| 36-60 | 39047 (50.83) | 36185 (47.11) | 34373 (44.75) |  | 109605 (47.56) |
| Mean ± SD | 35.86 ± 12.35 | 34.77 ± 12.89 | 34.59 ± 12.08 | <0.001 | 35.07 ± 12.46 |
| **Urbanization level** |  |  |  | <0.001 |  |
| 1 (highest) | 43307 (56.38) | 44391 (57.79) | 50043 (65.15) |  | 137741 (59.77) |
| 2 | 26857 (34.96) | 25085 (32.66) | 18727 (24.38) |  | 70669 (30.67) |
| 3 | 4108 (5.35) | 4913 (6.40) | 5240 (6.82) |  | 14261 (6.19) |
| 4 (lowest) | 112 (0.15) | 800 (1.04) | 914 (1.19) |  | 1826 (0.79) |
| unknown | 2432 (3.17) | 1627 (2.12) | 1893 (2.46) |  | 5952 (2.58) |
| **Insurance amount, NT$** |  |  |  | <0.001 |  |
| financially dependent | 508 (0.66) | 362 (0.47) | 453 (0.59) |  | 1323 (0.57) |
| 1-19999 | 27770 (36.15) | 26329 (34.28) | 25428 (33.10) |  | 79527 (34.51) |
| 20000-39999 | 34404 (44.79) | 35711 (46.49) | 32083 (41.77) |  | 102198 (44.35) |
| ≥40000 | 11666 (15.19) | 12161 (15.83) | 16604 (21.62) |  | 40431 (17.54) |
| unknown | 2468 (3.21) | 2253 (2.93) | 2249 (2.93) |  | 6970 (3.02) |
| **CCI score** |  |  |  | <0.001 |  |
| Mean ± SD | 1.13 ± 1.65 | 1.09 ± 1.67 | 1.07 ± 1.72 |  | 1.10 ± 1.68 |
| **Comorbidity** |  |  |  |  |  |
| Inflammatory disease of the ovary, fallopian tube, pelvic cellular tissue, and peritoneum | 12549 (16.34) | 12323 (16.04) | 10789 (14.05) | <0.001 | 35661 (15.47) |
| Inflammatory disease of the uterus | 3805 (4.95) | 3873 (5.04) | 4502 (5.86) | <0.001 | 12180 (5.29) |
| Inflammatory disease of the cervix, vagina, and vulva | 36221 (47.15) | 35147 (45.75) | 35562 (46.29) | <0.001 | 106930 (46.40) |
| Endometriosis | 2860 (3.72) | 3003 (3.91) | 2905 (3.78) | 0.149 | 8768 (3.80) |
| Hypertension | 13932 (18.14) | 12885 (16.77) | 11671 (15.19) | <0.001 | 38488 (16.70) |
| Diabetes mellitus | 9715 (12.65) | 8926 (11.62) | 8478 (11.04) | <0.001 | 27119 (11.77) |
| Hypertriglyceridemia | 128 (0.17) | 98 (0.13) | 60 (0.08) | <0.001 | 286 (0.12) |
| Hypercholesterolemia | 9370 (12.20) | 10162 (13.23) | 10239 (13.33) | <0.001 | 29771 (12.92) |
| Coronary artery disease | 7945 (10.34) | 7164 (9.33) | 6523 (8.49) | <0.001 | 21632 (9.39) |
| Disorders of eating | 94 (0.12) | 104 (0.14) | 104 (0.14) | 0.718 | 302 (0.13) |

SD, standard deviation; CCI score, Charlson Comorbidity Index score.
The tertile values, in ppm, were as follows: T1: < 2.17; T2: ≥ 2.17 and < 2.31; T3: ≥ 2.31.
The chi-squared test or one-way analysis of variance among tertiles of total hydrocarbons.
Urbanization level was defined at the beginning of the follow-up period.
Insurance amount was measured as the average value during the period of air pollutant exposure assessment.
Comorbidities were defined before the survival date.

**Table S1-10. Characteristics of the study population among tertiles of NMHC exposure**

| **Characteristic** | **Tertiles of average daily NMHC, n (%)** | | | **P value** | **Total (N = 230449)** |
| --- | --- | --- | --- | --- | --- |
|  | **T1 (lowest) (n = 76813)** | **T2 (n = 76819)** | **T3 (highest) (n = 76817)** |  |  |
| **Infertility** | 541 (0.70) | 1786 (2.32) | 2633 (3.43) | <0.001 | 4960 (2.15) |
| **Age, years** |  |  |  | <0.001 |  |
| 15-35 | 36591 (47.64) | 41096 (53.50) | 43157 (56.18) |  | 120844 (52.44) |
| 36-60 | 40222 (52.36) | 35723 (46.50) | 33660 (43.82) |  | 109605 (47.56) |
| Mean ± SD | 36.40 ± 12.38 | 34.60 ± 12.87 | 34.23 ± 12.01 | <0.001 | 35.07 ± 12.46 |
| **Urbanization level** |  |  |  | <0.001 |  |
| 1 (highest) | 43597 (56.76) | 43689 (56.87) | 50455 (65.68) |  | 137741 (59.77) |
| 2 | 24955 (32.49) | 27590 (35.92) | 18124 (23.59) |  | 70669 (30.67) |
| 3 | 5743 (7.48) | 3056 (3.98) | 5462 (7.11) |  | 14261 (6.19) |
| 4 (lowest) | 135 (0.18) | 733 (0.95) | 958 (1.25) |  | 1826 (0.79) |
| unknown | 2383 (3.10) | 1751 (2.28) | 1818 (2.37) |  | 5952 (2.58) |
| **Insurance amount, NT$** |  |  |  | <0.001 |  |
| financially dependent | 569 (0.74) | 316 (0.41) | 438 (0.57) |  | 1323 (0.57) |
| 1-19999 | 25043 (32.60) | 28853 (37.56) | 25631 (33.37) |  | 79527 (34.51) |
| 20000-39999 | 37106 (48.31) | 33138 (43.14) | 31954 (41.60) |  | 102198 (44.35) |
| ≥40000 | 11678 (15.20) | 12108 (15.76) | 16645 (21.67) |  | 40431 (17.54) |
| unknown | 2417 (3.15) | 2404 (3.13) | 2149 (2.80) |  | 6970 (3.02) |
| **CCI score** |  |  |  | <0.001 |  |
| Mean ± SD | 1.20 ± 1.72 | 1.09 ± 1.71 | 1.00 ± 1.61 |  | 1.10 ± 1.68 |
| **Comorbidity** |  |  |  |  |  |
| Inflammatory disease of the ovary, fallopian tube, pelvic cellular tissue, and peritoneum | 13082 (17.03) | 11889 (15.48) | 10690 (13.92) | <0.001 | 35661 (15.47) |
| Inflammatory disease of the uterus | 3813 (4.96) | 3713 (4.83) | 4654 (6.06) | <0.001 | 12180 (5.29) |
| Inflammatory disease of the cervix, vagina, and vulva | 36308 (47.27) | 34862 (45.38) | 35760 (46.55) | <0.001 | 106930 (46.40) |
| Endometriosis | 2754 (3.59) | 3139 (4.09) | 2875 (3.74) | <0.001 | 8768 (3.80) |
| Hypertension | 14552 (18.94) | 12534 (16.32) | 11402 (14.84) | <0.001 | 38488 (16.70) |
| Diabetes mellitus | 10280 (13.38) | 8856 (11.53) | 7983 (10.39) | <0.001 | 27119 (11.77) |
| Hypertriglyceridemia | 124 (0.16) | 107 (0.14) | 55 (0.07) | <0.001 | 286 (0.12) |
| Hypercholesterolemia | 10261 (13.36) | 9404 (12.24) | 10106 (13.16) | <0.001 | 29771 (12.92) |
| Coronary artery disease | 8537 (11.11) | 6912 (9.00) | 6183 (8.05) | <0.001 | 21632 (9.39) |
| Disorders of eating | 88 (0.11) | 115 (0.15) | 99 (0.13) | 0.160 | 302 (0.13) |

SD, standard deviation; CCI score, Charlson Comorbidity Index score.
The tertile values, in ppm, were as follows: T1: < 0.25; T2: ≥ 0.25 and < 0.33; T3: ≥ 0.33.
The chi-squared test or one-way analysis of variance among tertiles of non-methane hydrocarbon.
Urbanization level was defined at the beginning of the follow-up period.
Insurance amount was measured as the average value during the period of air pollutant exposure assessment.
Comorbidities were defined before the survival date.

**Table S1-11. Characteristics of the study population among tertiles of CH4 exposure**

| **Characteristic** | **Tertiles of average daily CH4, n (%)** | | | **P value** | **Total (N = 230449)** |
| --- | --- | --- | --- | --- | --- |
|  | **T1 (lowest) (n = 76816)** | **T2 (n = 72513)** | **T3 (highest) (n = 81120)** |  |  |
| **Infertility** | 335 (0.44) | 451 (0.62) | 4174 (5.15) | <0.001 | 4960 (2.15) |
| **Age, years** |  |  |  | <0.001 |  |
| 15-35 | 38313 (49.88) | 37390 (51.56) | 45141 (55.65) |  | 120844 (52.44) |
| 36-60 | 38503 (50.12) | 35123 (48.44) | 35979 (44.35) |  | 109605 (47.56) |
| Mean ± SD | 35.60 ± 12.38 | 35.04 ± 12.63 | 34.60 ± 12.36 | <0.001 | 35.07 ± 12.46 |
| **Urbanization level** |  |  |  | <0.001 |  |
| 1 (highest) | 41729 (54.32) | 45047 (62.12) | 50965 (62.83) |  | 137741 (59.77) |
| 2 | 28523 (37.13) | 18509 (25.53) | 23637 (29.14) |  | 70669 (30.67) |
| 3 | 3935 (5.12) | 6177 (8.52) | 4149 (5.11) |  | 14261 (6.19) |
| 4 (lowest) | 144 (0.19) | 1364 (1.88) | 318 (0.39) |  | 1826 (0.79) |
| unknown | 2485 (3.23) | 1416 (1.95) | 2051 (2.53) |  | 5952 (2.58) |
| **Insurance amount, NT$** |  |  |  | <0.001 |  |
| financially dependent | 469 (0.61) | 342 (0.47) | 512 (0.63) |  | 1323 (0.57) |
| 1-19999 | 29175 (37.98) | 25510 (35.18) | 24842 (30.62) |  | 79527 (34.51) |
| 20000-39999 | 33013 (42.98) | 32708 (45.11) | 36477 (44.97) |  | 102198 (44.35) |
| ≥40000 | 11532 (15.01) | 11932 (16.45) | 16967 (20.92) |  | 40431 (17.54) |
| unknown | 2627 (3.42) | 2021 (2.79) | 2322 (2.86) |  | 6970 (3.02) |
| **CCI score** |  |  |  | <0.001 |  |
| Mean ± SD | 1.10 ± 1.63 | 1.07 ± 1.61 | 1.13 ± 1.79 |  | 1.10 ± 1.68 |
| **Comorbidity** |  |  |  |  |  |
| Inflammatory disease of the ovary, fallopian tube, pelvic cellular tissue, and peritoneum | 12245 (15.94) | 10872 (14.99) | 12544 (15.46) | <0.001 | 35661 (15.47) |
| Inflammatory disease of the uterus | 3899 (5.08) | 4280 (5.90) | 4001 (4.93) | <0.001 | 12180 (5.29) |
| Inflammatory disease of the cervix, vagina, and vulva | 35936 (46.78) | 33596 (46.33) | 37398 (46.10) | 0.023 | 106930 (46.40) |
| Endometriosis | 2987 (3.89) | 2626 (3.62) | 3155 (3.89) | 0.008 | 8768 (3.80) |
| Hypertension | 13657 (17.78) | 12520 (17.27) | 12311 (15.18) | <0.001 | 38488 (16.70) |
| Diabetes mellitus | 9447 (12.30) | 8545 (11.78) | 9127 (11.25) | <0.001 | 27119 (11.77) |
| Hypertriglyceridemia | 115 (0.15) | 76 (0.10) | 95 (0.12) | 0.038 | 286 (0.12) |
| Hypercholesterolemia | 9286 (12.09) | 10148 (13.99) | 10337 (12.74) | <0.001 | 29771 (12.92) |
| Coronary artery disease | 7533 (9.81) | 6883 (9.49) | 7216 (8.90) | <0.001 | 21632 (9.39) |
| Disorders of eating | 92 (0.12) | 93 (0.13) | 117 (0.14) | 0.393 | 302 (0.13) |

SD, standard deviation; CCI score, Charlson Comorbidity Index score.
The tertile values, in ppm, were as follows: T1: < 1.93; T2: ≥ 1.93 and < 1.97; T3: ≥ 1.97.
The chi-squared test or one-way analysis of variance among tertiles of methane.
Urbanization level was defined at the beginning of the follow-up period.
Insurance amount was measured as the average value during the period of air pollutant exposure assessment.
Comorbidities were defined before the survival date.

Table S2. Pearson’s correlation analysis for air pollutants over the exposure period

|  | SO2 | CO | O3 | PM10 | PM2.5 | NOX | NO | NO2 | THC | NMHC | CH4 |
| --- | --- | --- | --- | --- | --- | --- | --- | --- | --- | --- | --- |
| SO2 | 1.000 | **0.161***** | **0.042***** | 0.622*** | 0.606*** | **0.229***** | **0.036***** | 0.409*** | **0.044***** | **0.131***** | **-0.086***** |
| CO |  | 1.000 | -0.637*** | -0.358*** | **-0.281***** | 0.970*** | 0.938*** | 0.902*** | 0.670*** | 0.851*** | **0.136***** |
| O3 |  |  | 1.000 | 0.403*** | 0.357*** | -0.607*** | -0.547*** | -0.608*** | -0.396*** | -0.482*** | **-0.102***** |
| PM10 |  |  |  | 1.000 | 0.937*** | -0.305*** | -0.478*** | **-0.090***** | -0.331*** | -0.408*** | **-0.085***** |
| PM2.5 |  |  |  |  | 1.000 | **-0.258***** | -0.450*** | **-0.028***** | -0.393*** | -0.406*** | **-0.199***** |
| NOX |  |  |  |  |  | 1.000 | 0.952*** | 0.946*** | 0.685*** | 0.841*** | **0.175***** |
| NO |  |  |  |  |  |  | 1.000 | 0.802*** | 0.769*** | 0.886*** | **0.269***** |
| NO2 |  |  |  |  |  |  |  | 1.000 | 0.524*** | 0.705*** | **0.057***** |
| THC |  |  |  |  |  |  |  |  | 1.000 | 0.836*** | 0.736*** |
| NMHC |  |  |  |  |  |  |  |  |  | 1.000 | **0.244***** |
| CH4 |  |  |  |  |  |  |  |  |  |  | 1.000 |

SO2, sulfur dioxide; CO, carbon monoxide; O3, ozone; PM10, particulate matter < 10 μm in size; PM2.5, particulate matter < 2.5 μm in size; NOX, nitrogen oxides; NO, nitrogen monoxide; NO2, nitrogen dioxide; THC, total hydrocarbons; NMHC, nonmethane hydrocarbons; CH4, methane.
***Correlation significant at the 0.001 level (two-tailed).

|Correlation coefficient values| <0.3 denote a low strength of correlation, which qualified as the controlling pollutant in multiple-pollutant models of targeted pollutants.

Table S3. Hazard ratios of long-term O3 exposure at a 1.73-ppb increment associated with the incidence of Infertility

| Population | Controlling pollutant | Adjusted HR (95% CI) | P value |
| --- | --- | --- | --- |
| Total (N = 232125) | - | 0.48 (0.47,0.49) | < 0.001 |
|  | SO2 | 0.38 (0.36,0.39) | < 0.001 |
|  | CH4 | 0.40 (0.38,0.41) | < 0.001 |
|  | SO2, CH4 | 0.40 (0.38,0.42) | < 0.001 |

HR, hazard ratio; CI, confidence interval; O3, ozone; SO2, sulfur dioxide; CH4, methane.
Additional pollutants were added into the pollutant models for multiple analysis only when Pearson’s correlation coefficient was <0.3.
Cox regression models were adjusted for age, urbanization level, insurance amount, CCI score, Inflammatory disease of the ovary, fallopian tube, pelvic cellular tissue, and peritoneum, Inflammatory disease of the uterus, Endometriosis, Hypertension, Diabetes mellitus, Hypertriglyceridemia, Hypercholesterolemia, Coronary artery disease, ambient temperature, lag0-2, season, and controlled pollutants (weak correlation with O3).

Table S4. Hazard ratios for incidence of infertility among women aged 15–45 years of long-term exposure at an SD increment.

| Pollutant | Adjusted HR (95% CI) | P value | SD |
| --- | --- | --- | --- |
| SO_2_ | 1.13 (1.09,1.17) | < 0.001 | 1.13 ppb |
| CO | 2.12 (2.06,2.19) | < 0.001 | 0.11 ppm |
| O_3_ | 0.48 (0.47,0.50) | < 0.001 | 1.73 ppb |
| PM_10_ | 1.36 (1.29,1.44) | < 0.001 | 8.93 μg/m^3^ |
| PM_2.5_ | 1.77 (1.67,1.88) | < 0.001 | 6.05 μg/m^3^ |
| NO_X_ | 1.77 (1.70,1.83) | < 0.001 | 6.80 ppb |
| NO | 1.64 (1.59,1.69) | < 0.001 | 3.68 ppb |
| NO_2_ | 1.73 (1.66,1.80) | < 0.001 | 3.48 ppb |
| THC | 2.11 (2.05,2.18) | < 0.001 | 0.12 ppm |
| NMHC | 1.49 (1.45,1.53) | < 0.001 | 0.08 ppm |
| CH_4_ | 2.84 (2.74,2.94) | < 0.001 | 0.07 ppm |
| HR, hazard ratio; CI, confidence interval; SD, standard deviation; SO_2_, sulfur dioxide; CO, carbon monoxide; O_3_, ozone; PM_10_, particulate matter < 10 μm in size; PM_2.5_, particulate matter < 2.5 μm in size; NO_X_, nitrogen oxides; NO, nitrogen monoxide; NO_2_, nitrogen dioxide; THC, total hydrocarbons; NMHC, nonmethane hydrocarbons; CH_4_, methane.  Cox regression models were adjusted for age, urbanization level, insurance amount, CCI score, Inflammatory disease of the ovary, fallopian tube, pelvic cellular tissue, and peritoneum, Inflammatory disease of the uterus, Inflammatory disease of the cervix, vagina, and vulva, Endometriosis, Hypertension, Diabetes mellitus, Hypertriglyceridemia, Hypercholesterolemia, Coronary artery disease, ambient temperature, lag0-2, season, and controlled pollutants | | | |

Table S5. The dose–response association between air pollutants and the risk of Infertility among women aged 15–45 years

| **Pollutant category** | **Tertiles of average daily pollutant** | **Population** | **Infertility** | **PY** | **Incidence Rate** | **Adjusted HR (95% CI)** | **P value** |
| --- | --- | --- | --- | --- | --- | --- | --- |
| SO2 | T1 (lowest) | Total (N = 176370) | 974 | 776111 | 12.55 | 1.00 (reference) | - |
|  | T2 |  | 1796 | 731829 | 24.54 | 1.23 (1.14,1.33) | < 0.001 |
|  | T3 (highest) |  | 2207 | 796537 | 27.71 | 1.71 (1.57,1.86) | < 0.001 |
| CO | T1 (lowest) | Total (N = 176370) | 442 | 730547 | 6.05 | 1.00 (reference) | - |
|  | T2 |  | 1351 | 781903 | 17.28 | 1.66 (1.48,1.85) | < 0.001 |
|  | T3 (highest) |  | 3184 | 792028 | 40.20 | 3.37 (3.02,3.76) | < 0.001 |
| O3 | T1 (lowest) | Total (N = 176370) | 3576 | 793206 | 45.08 | 1.00 (reference) | - |
|  | T2 |  | 795 | 786111 | 10.11 | 0.31 (0.28,0.34) | < 0.001 |
|  | T3 (highest) |  | 606 | 725159 | 8.36 | 0.24 (0.22,0.27) | < 0.001 |
| PM10 | T1 (lowest) | Total (N = 176370) | 1241 | 800341 | 15.51 | 1.00 (reference) | - |
|  | T2 |  | 1681 | 699285 | 24.04 | 1.37 (1.27,1.48) | < 0.001 |
|  | T3 (highest) |  | 2055 | 804852 | 25.53 | 2.04 (1.85,2.25) | < 0.001 |
| PM2.5 | T1 (lowest) | Total (N = 176358) | 830 | 805955 | 10.30 | 1.00 (reference) | - |
|  | T2 |  | 2077 | 730893 | 28.42 | 1.25 (1.15,1.36) | < 0.001 |
|  | T3 (highest) |  | 2062 | 767567 | 26.86 | 2.28 (2.05,2.55) | < 0.001 |
| NOX | T1 (lowest) | Total (N = 176370) | 602 | 725959 | 8.29 | 1.00 (reference) | - |
|  | T2 |  | 1777 | 779922 | 22.78 | 1.67 (1.52,1.84) | < 0.001 |
|  | T3 (highest) |  | 2598 | 798595 | 32.53 | 2.28 (2.05,2.53) | < 0.001 |
| NO | T1 (lowest) | Total (N = 176370) | 560 | 732767 | 7.64 | 1.00 (reference) | - |
|  | T2 |  | 1838 | 773408 | 23.76 | 1.71 (1.55,1.88) | < 0.001 |
|  | T3 (highest) |  | 2579 | 798302 | 32.31 | 2.50 (2.24,2.79) | < 0.001 |
| NO2 | T1 (lowest) | Total (N = 176370) | 674 | 723160 | 9.32 | 1.00 (reference) | - |
|  | T2 |  | 1505 | 783611 | 19.21 | 1.43 (1.30,1.57) | < 0.001 |
|  | T3 (highest) |  | 2798 | 797706 | 35.08 | 2.08 (1.89,2.29) | < 0.001 |
| THC | T1 (lowest) | Total (N = 175073) | 268 | 753432 | 3.56 | 1.00 (reference) | - |
|  | T2 |  | 1083 | 758343 | 14.28 | 1.14 (0.99,1.32) | 0.075 |
|  | T3 (highest) |  | 3605 | 775441 | 46.49 | 3.56 (3.09,4.10) | < 0.001 |
| NMHC | T1 (lowest) | Total (N = 175073) | 540 | 740103 | 7.30 | 1.00 (reference) | - |
|  | T2 |  | 1785 | 755412 | 23.63 | 1.27 (1.13,1.41) | < 0.001 |
|  | T3 (highest) |  | 2631 | 791701 | 33.23 | 1.78 (1.58,2.00) | < 0.001 |
| CH4 | T1 (lowest) | Total (N = 175073) | 335 | 755863 | 4.43 | 1.00 (reference) | - |
|  | T2 |  | 450 | 723490 | 6.22 | 1.06 (0.91,1.23) | 0.457 |
|  | T3 (highest) |  | 4171 | 807863 | 51.63 | 3.02 (2.67,3.41) | < 0.001 |
| PY, person years; HR, hazard ratio; CI, confidence interval; SO2, sulfur dioxide; CO, carbon monoxide; O3, ozone; PM10, particulate matter <10 μm in aerodynamic diameter; PM2.5, particulate matter <2.5 μm in aerodynamic diameter; NOX, nitrogen oxides; NO, nitric oxide; NO2, nitrogen dioxide; THC, total hydrocarbons; NMHC, non-methane hydrocarbon; CH4, methane.  The tertile values, in ppb (SO2, O3, NOX, NO, NO2); ppm (CO, THC, NMHC, CH4); and μg/m3 (PM10, PM2.5) were as follows:  SO2 (T1: < 3.59, T2: ≥ 3.59 and < 4.04, T3: ≥ 4.04); CO (T1: < 0.48, T2: ≥ 0.48 and < 0.59, T3: ≥ 0.59); O3 (T1: < 27.77, T2: ≥ 27.77 and < 29.11, T3: ≥ 29.11); PM10 (T1: < 47.66, T2: ≥ 47.66 and < 56.18, T3: ≥ 56.18); PM2.5 (T1: < 27.67, T2: ≥ 27.67 and < 34.95, T3: ≥ 34.95); NOX (T1: < 21.44, T2: ≥ 21.44 and < 29.60, T3: ≥ 29.60); NO (T1: < 4.93, T2: ≥ 4.93 and < 8.51, T3: ≥ 8.51); NO2 (T1: < 16.55, T2: ≥ 16.55 and < 20.49, T3: ≥ 20.49); THC (T1: < 2.17, T2: ≥ 2.17 and < 2.31, T3: ≥ 2.31); NMHC (T1: < 0.25, T2: ≥ 0.25 and < 0.33, T3: ≥ 0.33); CH4 (T1: < 1.93, T2: ≥ 1.93 and < 1.97, T3: ≥ 1.97).  per 10,000 person-years.  Cox regression models were adjusted for significantly unbalanced confounding factors, lag0-2, season, and ambient temperature. | | | | | | | |
